# Game Theory in Defence Applications: A Review

**DOI:** 10.3390/s22031032

**Published:** 2022-01-28

**Authors:** Edwin Ho, Arvind Rajagopalan, Alex Skvortsov, Sanjeev Arulampalam, Mahendra Piraveenan

**Affiliations:** 1 Faculty of Engineering, University of Sydney, Sydney, NSW 2006, Australia; edwin.ho.xo@gmail.com; 2 Weapons and Combat Systems Division, Defence Science and Technology (DST) Group, Adelaide, SA 5111, Australia; rajagopalan.arvind@gmail.com; 3 Maritime Division, Defence Science and Technology (DST) Group, Adelaide, SA 5111, Australia; alex.skvortsov@dst.defence.gov.au (A.S.); sanjeev.arulampalam@dst.defence.gov.au (S.A.)

**Keywords:** decision making, game theory, defence science, ground warfare, maritime warfare, aerial warfare, tracking, sensing

## Abstract

This paper presents a succinct review of attempts in the literature to use game theory to model decision-making scenarios relevant to defence applications. Game theory has been proven as a very effective tool in modelling the decision-making processes of intelligent agents, entities, and players. It has been used to model scenarios from diverse fields such as economics, evolutionary biology, and computer science. In defence applications, there is often a need to model and predict the actions of hostile actors, and players who try to evade or out-smart each other. Modelling how the actions of competitive players shape the decision making of each other is the forte of game theory. In past decades, there have been several studies that applied different branches of game theory to model a range of defence-related scenarios. This paper provides a structured review of such attempts, and classifies existing literature in terms of the kind of warfare modelled, the types of games used, and the players involved. After careful selection, a total of 29 directly relevant papers are discussed and classified. In terms of the warfares modelled, we recognise that most papers that apply game theory in defence settings are concerned with Command and Control Warfare, and can be further classified into papers dealing with (i) Resource Allocation Warfare (ii) Information Warfare (iii) Weapons Control Warfare, and (iv) Adversary Monitoring Warfare. We also observe that most of the reviewed papers are concerned with sensing, tracking, and large sensor networks, and the studied problems have parallels in sensor network analysis in the civilian domain. In terms of the games used, we classify the reviewed papers into papers that use non-cooperative or cooperative games, simultaneous or sequential games, discrete or continuous games, and non-zero-sum or zero-sum games. Similarly, papers are also classified into two-player, three-player or multi-player game based papers. We also explore the nature of players and the construction of payoff functions in each scenario. Finally, we also identify gaps in literature where game theory could be fruitfully applied in scenarios hitherto unexplored using game theory. The presented analysis provides a concise summary of the state-of-the-art with regards to the use of game theory in defence applications and highlights the benefits and limitations of game theory in the considered scenarios.

## 1. Introduction

Game Theory has become one of the conventional theoretical frameworks to model important decision making processes in many aspects of our life. The well-known examples can be found in economics, social sciences, finance, project management, computer science, civics, and epidemiology (see [1,2,3,4,5] and references therein). Since the seminal work of John Von Neumann, John Nash, and others [6,7,8], it has been well recognised that there is an optimal strategy in the context of complex interactions (games) between two or more parties (players) that can lead to a predictable outcome (payoff). In practical situations, this outcome can often be quantitative and amenable to arithmetic operations (cost, number of infected people, number of vaccinated people etc.), but often it can be qualitative in nature (such as risk, readiness level, health state etc.).

The application of game theory and related mathematical approaches have recently attracted ever-increasing attention in the defence domain. This is due to two driving factors. First, game theory provides a natural framework to promptly translate a high-level policy decision into optimal strategy by framing it in quantitative terms such as payoff, cost, gain or loss, risk, etc. This creates a united platform for defence decision-makers to support arriving at a particular decision. Second, it provides a rigorous mathematical framework for the evaluation and optimisation of numerous scenarios in accordance with predefined criteria. This prompt evaluation often becomes the critical success factor in the defence operational context, leading to decision superiority under time pressure. This also becomes the critical step in the development and deployment of various Artificial Intelligence (AI) capabilities in defence operations.

The application of game theory in defence has a sustained and diversified history ranging from the design of real-time military systems (e.g, applied in missile interception) to the support of strategic decisions on large defence investments and acquisitions. There is extensive literature on specific theoretical methods and tools and their defence applications. We believe that the review of this literature is of interest to the community dealing with operational analysis and data-driven decision support. This is the main motivation for the presented study.

Game theory [9,10] enhances military strategies and decision making processes with a holistic and quantitative analysis of situations [11]. For the military, the potential scenarios amenable to game-theoretic analysis include the rapidly growing applications of autonomous intelligent systems and game theory provides a comprehensive mathematical framework that greatly enhances the capabilities of decision making of the people who use these systems. Because of its potential, research into game theory is burgeoning, with more than a few papers beginning to emerge in the literature for this military research niche. This review aims to assist researchers in utilising the body of knowledge in game theory to develop smarter and safer decision making systems for defence practitioners. Given that the state of such research is still in an incipient phase, we do this by drawing connections between existing military knowledge with the nascent possibilities that game theory offers so that it can become a more widely understood and considered framework in military control systems.

To understand the state-of-the-art in the field of game-theoretic applications in defence, and to analyse the types of games used in such contexts, a review is needed. To the best of our knowledge, such a review, spanning different applications of game theory in a variety of military domains, is lacking. The goal of this paper is to present such a review that will provide a better understanding of the multitude of defence problems in which game theory can be successfully applied. Moreover, the multidimensional classification of the types of games used in different contexts will provide researchers insights into new ways of applying game theory in related problems. Finally, we present gaps in the literature which we hope will give rise to more research and development of novel game-theoretic approaches to defence problems.

Although it is not overly extensive, the body of literature around the game theory in the military has covered a notable portion of the different forms of engagement and combat. These papers cover past, present and future scenarios: from predictive strategies in potentially hostile situations to analytical assessments in hindsight of military standoffs thousands of years ago. Game theory has demonstrated the capacity to be useful in any such military scenario. However, rapid technological progress has led to consistently new frontiers of military engagement, each of these possessing its own complex systems. The overarching areas that have been addressed are Tracking systems (across all domains), Aerial combat, Ground combat, National Security issues, Cyberwarfare and Space systems. Notably, applications of game theory in Naval warfare have been few, and an exploration into the future research into areas like this will be discussed later in the review. Within each of these areas, there are a myriad of possibilities for new and innovative systems: different agents, different weapons, different control structures - and each of these could be enriched with game-theoretic analysis. While Haywood’s and Thunholm’s treatises on the game theory used in military decision making provide coverage of several different game types [12,13], there does not seem to be a paper that addresses the use of game theory in the military across each of the respective fields in the new context of military systems built on high-performance computing and complex algorithms. We aim to present the literature in such a way that it addresses all of the functions of game theory in military control systems in each key domain.

This review has considered in detail a total of 29 papers after careful selection. It highlights the scope and utility of each analysed paper by presenting it in terms of the essential game-theoretic concepts: players, game types, strategies and the key parameters of their payoff functions. It will act as both an annotated bibliography as well as a framework to understand and plan further research into the area. It will also lay out the fundamental tenets that are considered by players in every military decision-making scenario, as well as how they impact the decisions that are made by military personnel and systems, either while competing with hostile players or while cooperating with friendly players. This will make it possible for most military scenarios to be viewed as games and can provide, at the very least, an interesting new perspective on familiar military situations. The 29 papers reviewed here were selected from Scopus and Google Scholar, by a team of experts with related backgrounds from defence, academia and industry who could offer diverse perspectives, who identified the most pertinent papers based on diverse experience. Only papers written in English were considered. While it is acknowledged that an exhaustive search was not performed, the papers, to the best of our knowledge, cover a significant and representative section of the research niche we discuss here, and sufficiently demonstrate the trends, overlaps and gaps in the literature in this niche. It is confidently expected, therefore, that the presented analysis will provide a rigorous comparison between the analysed papers and highlight the strengths and weaknesses of each, while also highlighting the overall pros and cons of utilising game theory to model decision making in military contexts.

The rest of the paper is structured as follows. Section 2 will discuss the basic defence principles which are elaborated by the papers that we review, as well as introduce basic concepts of game theory. Section 3 investigates and analyses the literature and summarises the findings and associations in each of the papers. Section 4 elaborates our multi-dimensional classification of the literature based on the observations made in the previous section, and also presents citation and other metrics related to the papers reviewed. Section 5 identifies the gaps in the literature and based on this, highlights opportunities for future research in this niche, particularly areas of defence research that could benefit from the application of game theory where game theory has not been often applied so far. Section 6 provides an in-depth discussion about the utility of the findings and the presented review in general. Finally, Section 7 summarises our findings and classifications and provides broad conclusions.

## 2. Background

Ideologies, beliefs and knowledge about war have been shaping human knowledge and philosophy for centuries. The great works of Sun Tzu, Homer and Machiavelli [14,15,16] have not only established a foundation for knowledge etched into the essence of military decision making, but also provided insight into sociology and social psychology [17]. The military forms a core power bloc for many civilisations and is instrumental to both the growth of influence for existing nations, and the birth of new nations [18]. The military deals with conflicts in real-time, plans for the future, as well as reviews past engagements - and every single one of these activities has an impact on society [19]. This review therefore by necessity addresses many facets of military conflict across multiple physical domains, and the major decisions that need to be made in each of these domains will be summarised below. Across all of these domains, however, the value of targets, the value of resources and the priority of objectives are usually the key parameters that shape the payoff functions and strategies which in turn define the games that we use in modelling.

In this section, we discuss the concepts in defence science and technology, as well as game theory, which are necessary to understand and analyse the literature in the presented niche. First of all, let us consider the broad domains of defence and national security which are considered in this review. They can be summarised, as shown in Table 1.

As shown in Table 1, in this review, the focus is primarily on ‘command and control’ warfare, where decision making is critical. However, command and control warfare has applicability in traditional domains of warfare, such as land, sea, and air warfare, as well as modern domains of warfare, such as space and cyber warfare. At an orthogonal level, command and control warfare could also be sub-divided into Resource Allocation Warfare (RAW), Information Warfare (IW), Weapons Control Warfare (WCW), as well as Adversary Monitoring Warfare (AMW). Since these concepts are extensively used in our classification of literature, let us briefly introduce them first.

### 2.1. Warfare Types

1. **Resource Allocation Warfare (RAW)**: the allocation of military resources to achieve military objectives.
2. **Information Warfare (IW)**: the manipulation of information to achieve military objectives.
3. **Weapons Control Warfare (WCW)**: Control of weapons in achieving military objectives.
4. **Adversary Monitoring Warfare (AMW)**: Tracking the behaviour of an enemy to fulfil military objectives.

### 2.2. Warfare Domains

#### 2.2.1. Land Warfare

Technology is a dictating force of warfare, and technology is not as imperative to land warfare as it is to other domains [20]. The technology that has impacted land warfare has been relatively static and avoids the exposure of human resources if possible [21,22]. Interpersonal combat at a physical level is much less prevalent nowadays, making way for a greater focus on a positioning strategy. The literature which applied game theory to ground warfare includes a strong repository of Weapon-Target Allocation papers (which touch upon Weapon Control Warfare and Resource Allocation Warfare in the modern context), as well as papers that address ancient ground engagements and guerilla warfare. Where human lives are vulnerable, their protection is the most important element of these games, and the next priority is the protection of ground-based assets.

#### 2.2.2. Sea (Naval) Warfare

Given the importance of navies for the projection of power globally, there is a surprising paucity of publicly available literature on naval warfare—with or without the application of game theory. There is often mention of naval warfare in papers dealing with target tracking, but a discussion on military naval strategy is limited to outdated literature or discussion of bare essentials [23,24]. We will review the available papers in this regard, and highlight this as an area where there is a sizeable gap in the literature.

#### 2.2.3. Aerial Warfare

It was not long after the Wright Brothers invented the aeroplane that aerial warfare became a critical factor in combat and military campaigns [25]. In a combat medium rarely impeded by obstacles or dimensions, the nature of aerial combat is fast-paced, intuitive and incredibly treacherous, with unpredictable ‘rules’ for engagement [26,27]. In the present day, the factors to consider are vastly more complicated compared to a century ago, and there is no shortage of resources available to military forces to conduct aerial combat- both human and machine [28,29,30]. The literature shows that as a result of this abundance of arsenal, the intrinsic and potential value of both the targets and the resources used to engage is of particular importance in aerial warfare scenarios. Decisions about those values for both sides of the conflict need to be made when evaluating strategies for combat. As such, several papers deal with the use of game theory in aerial warfare.

#### 2.2.4. Cyber Warfare

Cybersecurity is the protection of IT systems and networks from being damaged/ disrupted/subjected to information theft. Cyberwarfare deals with the concept of Information and Communication Systems being deliberately attacked to obtain a military advantage. While Cybersecurity has been an important field in computer science for many decades now, literature about cyber warfare as such is more scarce, and in any case, heavily overlaps with applications of game theory in computer science in areas related to cybersecurity. This review presents and analyses some papers which are specifically concerned with cyber warfare.

#### 2.2.5. Space Warfare

While the notion of warfare in space has existed for almost a century, neither physical execution nor a body of theoretical strategies for space warfare has been established [31]. Nevertheless, this has not stopped military forces chasing the stars (literally and figuratively) [32,33] and has inevitably led to concepts from game theory being used in space warfare strategic thinking. This is currently mostly limited to satellite networks, where the key parameters of the game are optimised power use and signal strength across the network. The field is still quite young, and further military development in space seems to be inevitable, with which the corresponding literature dealing with applications of game theory in space warfare will also grow.

#### 2.2.6. Mixed/Other Warfare

Several papers address specific niches of defence applications of game theory, and yet cannot be classified as papers analysing a certain type of warfare. In some of these papers, the focus is more on the technology that is used: for example, target tracking. In others, the nature of the hostile actors against whom the defence needs to be conducted changes: for example, national security operations which target domestic terrorism threats rather than an opposing military force. Several papers deal with the use of game theory in such scenarios.

**Target tracking systems:** Target tracking in the military is the observation of a moving target and the surveillance of its position and manoeuvres [34,35,36]. Success in this domain relies on accuracy in the observed metrics and data, as well as efficient distribution and processing of all collected information [37]. With the advent of intelligent targets, the military must also incorporate predictive methods to maintain ideal tracking performance. The literature reviewed in this regard covers topics from tracking strike missiles to theatre ballistic missiles and tracking unknown intelligent agents to enemy aircraft. Key considerations in this area that shape the games played involve whether or not the target is ‘intelligent’/can take evasive action, whether or not the target will have an optimal trajectory, and whether or not the target will have defenders [38]. Target tracking applications of game theory mostly occur in aerial and naval warfare, including underwater surveillance.

**National Security applications**: Game theory often finds application in national security and anti-terrorism related fields. This includes predicting and preparing for terrorist attacks, as well as resource allocation scenarios for the protection of key personnel and landmarks/other potential targets for terrorist activity. While the value of potential targets and the likelihood of attacks are obviously key parameters governing the payoff functions of games in this niche, the subsequent social, economic and political ramifications are equally instrumental in modelling games in this area [39,40]. Few military conflicts have as much exposure as those on the home front [41], and the fallout from terrorist attacks and their effect on public mood and confidence in the security apparatus are often taken into account in modelling payoff functions in this area.

### 2.3. Game Theory

Game theory, which is the study of strategic decision making, was first developed as a branch of microeconomics [6,10,42,43]. However, later it has been adopted in diverse fields of study, such as evolutionary biology, sociology, psychology, political science, project management, financial management and computer science [1,2,5,9,11,44]. Game theory has gained such wide applicability due to the prevalence of strategic decision-making scenarios across different disciplines. Game Theory provides insight into peculiar behavioural interactions like the cooperative interactions within groups of animals [45], the bargaining and exchange in a marriage [46] or the incentivisation of Scottish salmon farmers [47]. A game typically consists of two or more players, a set of strategies available to these players, and a corresponding set of payoff values (also referred to as utility values) for each player (which are usually presented as a payoff-matrix in the case of two-player games) [44,48].

#### 2.3.1. Pure vs. Mixed Strategies

A pure strategy in a game provides a complete definition of how a player will play a game. A player’s strategy set is the set of pure strategies available to that player [10].

A mixed strategy is a combination of pure strategies where a particular probability *p* (where 0≤p≤1) is associated with each of these pure strategies. Since probabilities are continuous, there are infinitely many mixed strategies available to a player. A totally mixed strategy is a mixed strategy in which the player assigns a strictly positive probability to every pure strategy. Therefore, any pure strategy is actually a degenerate case of a mixed strategy, in which that particular strategy is selected with probability 1 and every other strategy is selected with probability 0.

#### 2.3.2. Nash Equilibrium

The concept of Nash equilibrium is fundamental to game theory. It is a state (a set of strategies) in a strategic game from which no player has an incentive to deviate unilaterally, in terms of payoffs. Both pure strategy and mixed strategy Nash equilibria can be defined. A strategic game can often have more than one Nash equilibrium [7]. Every game with a finite number of players in which each player can choose from finitely many pure strategies has at least one Nash equilibrium in mixed strategies, it is proven [7].

The formal definition of Nash equilibrium is as follows. Let (S,f) be a game with *n* players, where $S_{i}$ is the strategy set of a given player *i*. Thus, the strategy profile *S* consisting of the strategy sets of all players would be, $S=S_{1}$ × $S_{2}$ × $S_{3}\ldots$ × $S_{n}$. Let $f\left(x\right)=(f_{1}\left(x\right),\ldots ,f_{n}\left(x\right))$ be the payoff function for strategy set x∈S. Suppose $x_{i}$ is the strategy of player *i* and $x_{-i}$ is the strategy set of all players except player *i*. Thus, when each player i∈1,…,n chooses strategy $x_{i}$ that would result in the strategy set $x=(x_{1},\ldots ,x_{n})$, giving a payoff of $f_{i}\left(x\right)$ to that particular player, which depends on both the strategy chosen by that player ($x_{i}$) and the strategies chosen by other players ($x_{-i})$. A strategy set $x^{*}\in S$ is in Nash equilibrium if no unilateral deviation in strategy by any single player would return a higher utility for that particular player [10]. Formally put, $x^{*}$ is in Nash equilibrium if and only if:

(1) $$\forall i,x_{i}\in S_{i}:f_{i}\left(x_{i}^{*},x_{-i}^{*}\right)\geq f_{i}\left(x_{i},x_{-i}^{*}\right)$$

#### 2.3.3. Non-Cooperative Games and Cooperative Games

Typically, games are taken to be played for the self-interest of the players, and even when the players cooperate, that is because cooperation seems to them as the best strategy under the circumstances to maximise the individual payoffs of the players. In such games, the cooperative behaviour, if it emerges, is driven by selfish goals and is transient. These games can be termed ’non-cooperative games’. These are sometimes referred to, rather inaccurately, as ‘competitive games’. Non-cooperative game theory is the branch of game theory that analyses such games. On the other hand, in a cooperative game, sometimes also called a coalitional game, players form coalitions, or groups, sometimes due to external enforcement of cooperative behaviour, and competition, if it emerges, takes place between these coalitions [7,8,9]. Cooperative games are analysed using cooperative game theory, which predicts which coalitions will form and the payoffs of these coalitions. Cooperative game theory focuses on surplus or profit-sharing among the coalition [49], where the coalition is guaranteed a certain amount of payoff by virtue of the coalition being formed. Often, the outcome of a cooperative game played in a system is equivalent to the result of a constrained optimisation process [50].

#### 2.3.4. Zero-Sum Games

Zero-sum games are a class of competitive games where the total of the payoffs of all players is zero. In two-player games, this implies that one player’s loss in the payoff is equal to another player’s gain in the payoff. A two-player zero-sum game can therefore be represented by a payoff matrix that shows only the payoffs of one player. Zero-sum games can be solved with the mini-max theorem [51], which states that in a zero-sum game there is a set of strategies that minimises the maximum losses (or maximises the minimum payoff) of each player. This solution is sometimes referred to as a ‘pure saddle point’. It can be argued that the stock market is a zero-sum game. In contrast, most valid economic transactions are non-zero-sum since each party considers that, what it receives is more valuable (to itself) than what it parts with [10].

#### 2.3.5. Perfect vs. Imperfect Information Games

In a perfect information game, each player is aware of the full history of the previous actions of all other players, as well as the initial state of the game. In imperfect information games, some or all players do not have access to the entirety of information about other players’ previous actions [52,53].

#### 2.3.6. Simultaneous Games and Sequential Games

A simultaneous game is either a normal-form game or an extensive-form game where on each iteration, all players make decisions simultaneously. Therefore, each player is forced to decide without knowing about the decisions made by other players (on that iteration). On the contrary, a sequential game is a type of extensive-form game where players make their decisions (or choose their strategies) in some predefined order [52,53]. For example, a negotiation process can be modelled as a sequential game if one party always has the privilege of making the first offer, and the other parties make their offers or counteroffers after that. In a sequential game, at least some players can observe at least some of the actions of other players before making their own decisions (otherwise, the game becomes a simultaneous game, even if the moves of players do not happen simultaneously in time). However, it is not a must that every move of every previous player should be observable to a given player. If a player can observe every move of every previous player, such a sequential game is known to have ‘perfect information’. Otherwise, the game is known to have ‘imperfect information’ [10].

#### 2.3.7. Differential Games

Differential Games are often extensive form games, but instead of having discrete decision points, they are modelled over a continuous time frame [10]. In such games, each state variable evolves continuously over time according to a differential equation. Such games are ideal for modelling rapidly evolving defence scenarios where each player engages in selfish optimisation of some parameter. For example, in missile tracking problems, the pursuer and the target both try to control the distance between them, whereas the pursuer constantly tries to minimise this distance and the target constantly tries to increase it. In such a scenario, iterative rounds of decision making are much too discrete to model the continuous movements and computations of each player. Differential games are ideal to model such scenarios.

#### 2.3.8. Common Interest Games

Common interest games are another class of non-cooperative games in which there is an action profile that all players strictly prefer over all other profiles [52]. In other words, in common interest games, the interests of players are perfectly aligned. It can be argued that common interest games are the antithesis of zero-sum games, in which the interests of the players are perfectly opposed so that any increase in fortune for one player must necessarily result in the collective decrease in fortune for others. Common interest games were first studied in the context of cold war politics, to understand and prescribe strategies for handling international relations [54]. Therefore, it makes sense to classify non-cooperative games into common interest games and non-common interest games, just as much as it makes sense to classify them into zero-sum games and non-zero-sum games, as these two concepts (zero-sum games and common interest games) represent extreme cases of non-cooperative games.

#### 2.3.9. Signaling Games

A signalling game [52] is an incomplete information game where one player has perfect information and another does not. The player with perfect information (the Sender *S*) relays messages to the other player (the Receiver *R*) through signals, and the other player will act on those signals after inferring the information hidden in the messages. The Sender *S* has several potential *types*, of which the exact type *t* in the game is unknown to the Receiver *R*. *t* determines the payoff for *S*. *R* has only one type, and that payoff is known to both players.

The game is divided into the sending stage and the acting stage. *S* will send one of $M=\{m_{1},m_{2},m_{3},\ldots ,m_{j}\}$ messages. *R* will receive that message and respond with an action from a set $A=\{a_{1},a_{2},a_{3},\ldots ,a_{k}\}$. The payoff that each player receives is determined by the combination of the Sender’s type and message, as well as the action that the Receiver responds with. An example of the signalling game is the Beer-Quiche Game [52], in which Player B, the receiver, chooses whether or not to duel Player A. Player A is either surly or wimpy, and Player B would only like to duel the latter. Player A chooses to have either a Beer or a Quiche for breakfast. While they prefer a quiche, a quiche signals information from a stereotype that quiche eaters are wimpy. Player B must analyse how each decision, duel or not duel, will give them a better payoff depending on which breakfast Player A chooses.

#### 2.3.10. Behavioural Game Theory

Behavioural Game Theory combines classical game theory with experimental economics and experimental psychology, and in doing so, relaxes many simplifying assumptions made in classical game theory which are unrealistic. It deviates from simplifying assumptions such as perfect rationality [55], the independence axiom, and the non-consideration of altruism or fairness as motivators of human decision making [56,57]. We will show in this review that the approaches related to behavioural game theory are crucial in modelling military scenarios, such as in signalling games.

#### 2.3.11. Evolutionary Game Theory

Evolutionary game theory is an outcome of the adoptation of game theory into the field of evolutionary biology [58]. Some of the critical questions asked in evolutionary game theory include: which populations/strategies are stable? which strategies can ‘invade’ (become popular) in populations where other strategies are prevalent? How do players respond to other players receiving or perceived to be receiving better payoffs in an iterated game setting? etc. Evolutionary games are often modelled as iterative games where a population of players play the same game iteratively in a well-mixed or spatially distributed environment.

A strategy can be identified as an evolutionary stable strategy (ESS) if, when prevalent, it has the potential to prevent any mutant strategy from percolating its environment. Alternatively, an ESS is the strategy that, if adopted by a population in a given environment, cannot be invaded by any alternative strategy. Hence, there is no benefit for a player to switch from an ESS to another strategy. Therefore, essentially, an ESS ensures an extended Nash equilibrium. For a strategy $S_{1}$ to be ESS against another ‘invading’ strategy $S_{2}$, one of the two conditions mentioned below needs to be met, in terms of expected payoff *E*.

1. $E\left(S_{1},S_{1}\right)>E\left(S_{2},S_{1}\right)$: By unilaterally changing strategy to $S_{2}$, the player will lose out against another player who sticks with the ESS $S_{1}$.
2. $E\left(S_{1},S_{1}\right)=E\left(S_{2},S_{1}\right)$ & $E\left(S_{1},S_{2}\right)>E\left(S_{2},S_{2}\right)$: a player, by converting to $S_{2}$, neither gains nor loses against another player who sticks with the ESS $S_{1}$, but playing against a player who has already ‘converted’ to $S_{2}$, a player is better off playing the ESS $S_{1}$.

If either of these conditions are met, the new strategy $S_{2}$ is incapable of invading the existing strategy $S_{1}$, and thus, $S_{1}$ is an ESS against $S_{2}$. Evolutionary games are typically modelled as iterative games, whereby players in a population play the same game iteratively [52].

#### 2.3.12. Other Recent Advances in Game Theory

It should be noted that there are several other branches of game theory that were not mentioned in the above sub-sections, and there have been also several recent advances that have not been mentioned. Game theory is used in increasingly more diverse scenarios and applications. For example, game theory has been used to determine the market share of competitors in the telecommunication industry [59], or implementation and construction of biogas plants [60]. In some applications, payoffs of matrix games are constructed to contain fuzzy elements, which, it is argued, makes the modelled scenarios more realistic [61,62]. Similarly, quantum game theory is an emerging field [63,64], which introduces superposed initial states, quantum entanglement of initial states, and superposition of strategies. Not all such advances can be summarised here. Therefore, this section has provided a basic introduction to only those game-theoretic concepts which are often used in the defence literature, and particularly in the papers that we review. Therefore, to the reader unfamiliar with game theory, the above subsections presented an elementary introduction. Please see [10,52] for more elaborate treatments of the concepts presented.

With this background, we now review the available literature which deals with the applications of game theory in defence science and technology.

## 3. Use of Game Theory in Defence Science and Technology Applications

As mentioned earlier, the primary parameters that influence the payoff matrix in games modelling defence scenarios are the value of targets, the value of resources and the priority of objectives. Other than this, the games used in defence applications can vary greatly, as we will see below. For this reason, this section is structured based on the domain (type of warfare) each paper covers. Where a paper covers more than one domain, it is included in the most relevant subsection/domain. We however analyse in detail the type of games used, the way the payoff functions were structured, the available strategies and equilibria etc for each paper.

### 3.1. Papers Dealing with Land Warfare

In Land warfare related applications of game theory, most studies focus on defensive warfare, whereby the military makes decisions on how to best allocate their ground defences to multiple threats. Some studies also focus on historical land-based conflicts and provide game-theoretical analysis in hindsight, revealing how some decisions made from intuition in historical conflicts had a rational and mathematical justification. Land warfare can result in very heavy casualties, so understanding how to best minimise human losses is a key component (though not the only objective) of land warfare. Quite often, prioritising military resources is also fundamental to success and often features prominently in strategic decisions. Furthermore, often in scenarios involving ground warfare, it is important to assess the knowledge about opponents, their possible tactics, or terrain: it may become necessary to combat airborne forces being inserted at certain places, or it may be needed to traverse uncertain territory. In each of these situations, understanding where a force has imperfect information will help that force to make rational decisions.

Several papers use game theory to model land warfare in current and historical contexts. Bier et al. [65] design a game to best assign defensive resources to a set of locations/resources that need to be protected. The attacker must then decide how they choose to split their force to attack the different targets. The game is modelled as a two-player game of normal form. The payoff in this game is absolute, and an attack on a location *i* is either a success or a failure, where the attacker gains $a_{i}$ and the defender loses $d_{i}$. Since orders for an attack are confirmed ahead of an attack, attackers must use a set of pure strategies. The game can be played both simultaneously or sequentially. That is, the game can be played depending on whether or not the attacker knows how the defender has assigned their resources before making their decision. This leads to the ideal strategy of leaving some targets undefended and strengthening defences in key areas by leaving some areas vulnerable.

The next paper we review is Gries et al. [66] which is a comprehensive investigation into the utility of game theory principles in guerilla/destabilisation warfare. The significant factors they model are: destabilisation insurgents often attack randomly, creating a continuous threat that must have a continuous mitigation and detection strategy; the duration of a war is important to consider, and will change the value that is assigned to targets and assets; time preferences play a critical role in setting priorities, as judgements of value determine strategic decisions which in turn determine success or failure. The game model they propose involves both a sequential non-cooperative game and a simultaneous non-cooperative game, in each of which the two players are the guerilla force and the government. For these conflicts, the economic and social impacts are much more significant than military losses and gains and therefore play a much more significant role in calculating the value of outcomes.

**Figure 1:**
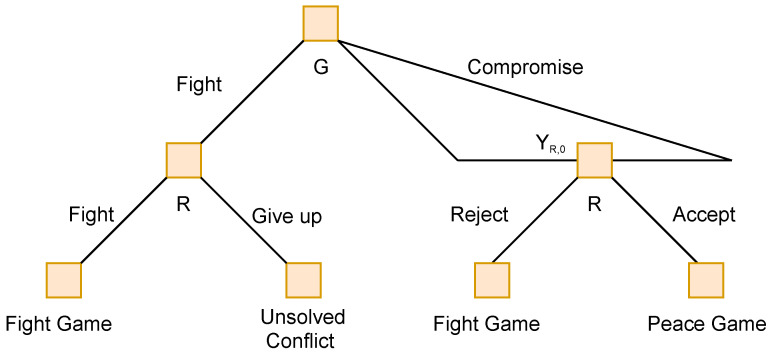
Destabilisation Warfare game [66], where the government and rebel decision points are highlighted.

The game specifically models moments when each side looks to try and find peace or conflict with the other. At each of these moments, the government forces must consider the financial cost of each option, while the rebels examine the order of priority of the engagements, and what portion of their fighting force they will make available for each engagement. Figure 1 demonstrates an example of the decision tree to emerge from these moments in destabilisation warfare, where G represents the Government decisions and R represents the Rebel decisions.

Krisnamurthy et al. [67] investigate game-theoretic control of dynamic behaviour of an unattended ground sensor network (UGSN) to acquire information about intruders. Each sensor in this network is capable of receiving measurements, with specified accuracy, of the range and bearing of nearby targets which they then transmit to a local hub for data fusion. In this framework, while more sensor measurements and larger volumes of transmission of measurements may lead to better target awareness, this also results in the undesirable effect of greater consumption of limited battery power. Hence, the goal to which game theory is applied is to optimally trade-off target awareness, data transmission and energy consumption using a two-time scale, hierarchical approach.

The authors demonstrate that the sensor activation and transmission scheduling problem can be decomposed into two coupled decentralized algorithms. In particular, the sensors are viewed as players in a non-cooperative game and an adaptive learning strategy is proposed to activate the sensors according to their proximity to targets of interest. This turns out to be a correlated equilibrium solution of this non-cooperative game. Next, the transmission scheduling problem, in which each sensor has to decide at each time instant, whether to transmit data and waste battery power or to wait and increase delay, is formulated as a Markov Decision process with a penalty terminal cost. The main result of this formulation is to show that the optimal transmission policy has a threshold structure which is then proved using the concept of supermodularity.

There are several studies that analyse historical conflicts, which occurred predominantly on land, using a game-theoretic prism. For example, Cotton and Liu [68] describe two ancient Chinese military legends and model them as signalling games. In both games, legendary military leaders are faced with formidable opposing armies with much greater numbers and strength than their own, but instead of retreating, they prepare to engage, acting as if they are setting up for an ambush. Their opponents with imperfect information are left only with the messages they can infer from their opponents’ actions; spooked by the perceived confidence and the reputation that these generals carried, the opposing armies, though in actuality are of superior strength, choose not to engage. Through a brave and ingenious bluff, both generals achieve an equilibrium solution in their favour by standing their ground. They do this by creating deception without direct communication, which follows the template of the aforementioned Beer-Quiche signalling game.

**Figure 2:**
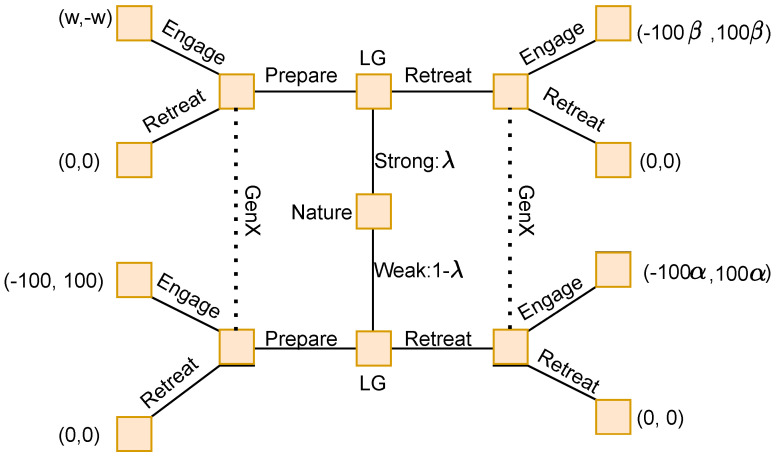
The 100 Horsemen signaling game [68].

The first game described by Cotton and Liu is the “100 Horsemen” game. They describe a piece of history where a hundred Han horsemen travelling alone encounter a large Xiongnu force numbering in the thousands. Their available strategies are to retreat or engage. If they retreat, and the enemy engage, they will very likely be run down and defeated; if they engage and the enemy also engage, they will be eliminated in battle. The best outcome for them is to somehow force an enemy retreat. The enemy is uncertain if the horsemen are travelling with a greater army. They see the horsemen move to engage, and decide not to take the risk, and retreat. The situation is translated into a two-player game, with two strategies. It is represented in the Figure 2 below:

In Figure 2:

LG represents the decision point for General Li Guang, of the Han forces.

GenX represents the decision point for the opposing Xiongnu force.

Payoffs are listed as (LG, GenX)

λ∈(0,1) represents the Generals’ ability, as either strong or weak

α and β represent the proportion of Han horsemen killed in a retreat

*w* is a positive parameter

**Figure 3:**
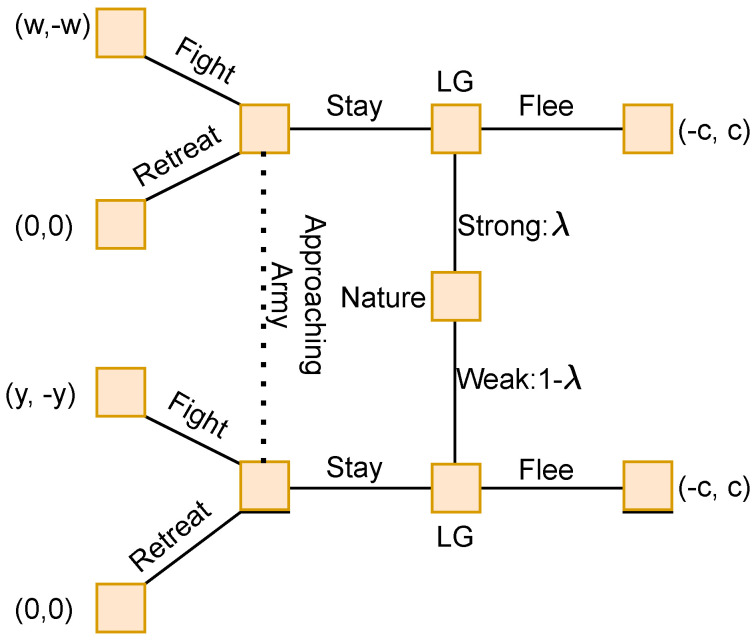
The Empty City signaling game [68].

The second game is very similar to the first. In this game, a small city is guarded by the formidable General Zhuge Liang. He learns that a great hostile army is approaching the city. He is faced with two options. He could run, after which he would secede the city and likely be chased down by the approaching army, or he could stay and defend the city. If he chose the latter, and the army were to engage, he would likely lose his life, his army and the city. Faced with this dilemma, he orders his men to hide out of sight, so that the city appears empty from the outside. He climbs to the top of the foremost tower of the city and plays music. The opposing general, aware of General Liang’s experience and prowess, suspects that the General has taken this unassuming position in the tower in the empty city to ambush his army, and they move away from the city to avoid being ambushed. General Liang sent effectively two signals here. The first was his reputation, a signal encompassing his strategic and military strength. The second was his choice to stay and defend the city. With these two pieces of information, and nothing else about the whereabouts or magnitude of General Liang’s army, the opposing army chooses the safe option of zero loss and leaves. This piece of history is modelled as another two-player signalling game, shown in Figure 3 below:

In Figure 3:

ZL represents the decision point for the General Zhuge Liang

Payoffs are listed as (ZL, Opposing Army)

λ∈(0,1) represents the Generals’ ability, as either strong or weak

*c* represents the value of the city

*w* represents the gains if ZL’s army matched the opposing army

*y* represents the losses if ZL’s army is weaker than the opposing army, and y>c since it encompasses losing the city

Both pieces of history represent distinguished military decision making in the face of near-certain defeat, and are in fact examples of Generals with a strong understanding of the nuances of signals and rational decision making in strategic interactions forcing an outcome that is favourable to themselves.

### 3.2. Papers Dealing with Naval Warfare

Surprisingly, papers that directly and primarily deal with naval warfare by using game theory are comparatively rare, even though naval warfare predates aerial warfare in human history by a considerable margin. Levin [69] studies aspects of naval warfare in the previous centuries using concepts from game theory. In the 18th and 19th centuries, the powerful nations of the time built warships with cannons positioned along their sides. It meant that ships could attack typically only to their sides. When sailing as an armada, the standard approach was to form a ‘line of battle’ i.e., a column of allied naval ships sailing in a direction such that their sides would face the enemy, also positioned in a line. The two parallel opposing fleets could then attack each other with a large number of cannons. The ‘line of battle’ strategy is considered to be a Nash-equilibrium because neither fleet would gain from performing raking (a tactic of the era, whereby an attacking ship would attempt to sail across its adversary’s stern, concentrating cannon fire there while the enemy could only respond minimally due to having fewer cannon placements in the stern. The attacking ship would damage both the stern and some of the broadsides of its adversary). According to Levin [69] raking was not preferred in a fleet as this would mean having to first sail ahead of its enemy and then turn towards it—a challenging task when the ships’ speeds were roughly equal and manoeuvring was difficult. As neither fleet would gain from turning towards the enemy and neither would get ahead, Levine concludes that this strategy—forming a line of battle and sailing parallel to the other fleet—was each fleet’s best response, and thus represented a Nash equilibrium.

Levine goes on to mention battles in which English fleets deviated from the above strategy and sailed orthogonally towards a French and Franco-Spanish fleet. In the first battle Levine mentions, it was likely unplanned. In the second—the 1805 Battle of Trafalgar—it was by careful design: the English fleet divided itself into two columns, each of which sailed orthogonally to the Franco-Spanish line, raking fire for about 45 minutes before crashing through it and beginning a general melee. The English would go on to isolate the middle of the Franco-Spanish fleet to score a decisive victory. Levine considers both battles to be counterexamples to his thesis. However, in the Battle of Trafalgar, it is possible that the English strategy was a best response to the likely Franco-Spanish strategy of forming an orthodox line of battle. The English admiral, Lord Nelson, desired to keep the Franco-Spanish fleet from escaping—which they could if both fleets formed parallel lines of battle—thus reducing the reward he would get for forming his own fleet into a line of battle. Moreover, he may have estimated that the poor gunnery of the French and Spanish ships would lessen the effect of the raking fire, thus reducing the negative reward he would get for directly charging the Franco-Spanish fleet. In his eyes, this may have made the unorthodox option a better response to the likely Franco-Spanish strategy than the orthodox line of battle. While Levine did not explicitly attribute these strategies in naval battles of the era to game theory, the adopted strategies could nonetheless be justified by game-theoretic analysis: an example of ’intuitive’ application of game theory without formally studying it.

Maskery et al. 2007 (a) [70] study the problem of deploying counter-measures against anti-ship missiles using a network-enabled operations (NEOPS) framework, where multiple ships communicate and coordinate to defend against a missile threat. Here, the missile threats are modelled as a discrete Markov process and they appear at random positions within a fixed physical space and move towards the ships obeying some known target dynamics and guidance laws. The ships which are equipped with counter-measures (CM) such as decoys and electromagnetic jamming signals are modelled as the players of a transient stochastic game, where the actions of the individual players include the use of CM to maximize their own safety while cooperating with other players which are essentially aiming to achieve the same objective. The optimal strategy of this game-theoretic problem is a correlated equilibrium strategy and is shown to be achieved via an optimization problem with bilinear constraints. This is in contrast to the Nash equilibrium solution proposed tepmaskery2007decentralized to a related problem but one without player coordination. A noteworthy contribution of this paper is that it also quantifies the amount of communication necessary to implement the NEOPS equilibrium strategy. This paper highlights the utility of game-theoretic methods in analysing optimal strategies in network-enabled systems which are critical in modern warfare.

In [71], Maskery et al. 2007 (b) consider the problem of network-centric force protection of a task group against anti-ship missiles. The decision-makers in this model are the ships equipped with hard-kill/soft-kill weapons (counter-measures) and these ships are also considered the players in the formulation of this problem in a game-theoretic setting. The platforms must make critical decisions independently on the optimal deployment of the counter-measures while they simultaneously work towards a common goal of protecting the members of the task group. Essentially this is a decentralised missile deflection problem in a naval setting which is formulated as a transient stochastic game for which the ships may compute a joint counter-measure policy that is in Nash equilibrium. Here, the ships play a game with each other instead of with a missile. This approach naturally lends itself to decentralised solutions which may be implemented when full communication is not feasible. Moreover, this formulation leads to an interpretation of the problem as a stochastic shortest past game for which Nash Equilibrium solutions are known to exist.

Bachmann et al. [72] analyse the interaction between radar and jammer using a noncooperative two-player, zero-sum game. In their approach, the radar and jammer are considered ‘players’ with opposing goals: the radar tries to maximize the probability of detection of the target while the jammer attempts to minimize its detection by the radar by jamming it. Bachmann et al. [72] assume a Swerling Type II target in the presence of Rayleigh distributed clutter, for which certain utility functions are described for cell-averaging (CA) and order-statistic (OS) CFAR processors in different cases of jamming. This game-theoretic formulation is solved by optimizing these utility functions subject to constraints in the control variables (strategies), which for the jammer are jammer power and the spatial extent of jamming while for the radar the available strategies include the threshold parameter and reference window size. The resulting matrix-form games are solved for optimal strategies of both radar and jammer from which they identify conditions under which the radar and jammer are effective in achieving their individual goals.

### 3.3. Papers Dealing with Aerial Warfare

Aerial combat is often a normal-form game where decisions about utilised resources are made before the engagement, based on assumptions and knowledge about the strength of different elements of the arsenal. For example, Suppression of Enemy Air Defense vehicles (SEADs) are effective against ground-to-air defences and Surface to Air Missiles (SAMs), but will not be useful against fighter aircraft. Therefore, when military personnel decide which resources to use in an engagement, they need to weigh how valuable each of their resources is, as well as how important the objective is to both sides of the conflict. If the attacking force values a target much more than it is actually worth, then their increased resource expenditure may be detrimental to their military campaign as a whole. With humans operating the aerial weaponry usually, their respective abilities and skillsets, and the likelihood of them executing their mission, need to be considered.

There is limited literature on aerial combat modelled with game theory. Hamilton [73] provides a comprehensive guide to applications of game theory on multiple Aerial warfare situations. Hamilton suggests using game theory to devise strategies not only based on one’s own military options but also expectations around enemy actions as well. Game theory accounts for different interactions with the enemy, rather than simply considering which side had superior maximum-effort power. Nowadays, many military forces can adapt to instantly changing situations and adjust their actions based on those new circumstances. As such, Hamilton suggests first determining all of the tactical options available to each side. As stated earlier, one of the most fundamental elements of using game theory for the military is understanding exactly how much value each asset holds—and detailing the inventory and strategic possibilities of both sides will best clarify all strategic options. For each option, Hamilton suggests assigning a numerical value—a Measure of Effectiveness (MoE). Decisions about MoEs are important because being accurate with MoEs will underpin the choices that are made strategically. Incorrect MoEs can lead to incorrect strategic decisions, and perhaps also result in poor understanding of why the decision was wrong. An example of this (although not in the aerial warfare context) was the Vietnam War, where the early US strategy was maximising the neutralisation of Viet Cong soldiers. Since the Northern Vietnamese leadership did not place great emphasis on their infantry, the US strategy ultimately led to a loss in the war. Next, Hamilton suggests calculating the combined value for all possible interactions between strategies of both sides of the conflict. This will generate a matrix of payoffs, from which it is possible to derive the optimum or dominant strategy for each player, and then an equilibrium solution. Thus, ahead of any engagement a military leader may partake in, they have a well-formed idea of the expected result of the game. A caveat that Hamilton adds to these guidelines is to consider the length of a military campaign as a whole. The values that can be assigned to a resource for one battle or strike attack may be small if they are cheap to replace or large in number. However, depending on the number of such skirmishes throughout a campaign, those resources may become pivotal.

To illustrate these points, Hamilton applies them to a standard aerial warfare game of SEADs and time-critical targets. In this combat, the ’Blue’ side is trying to eliminate some ground-based targets. To do this, they use SEADs. In response, the ’Red’ side will fire SAMs, which SEADs struggle to avoid. However, expecting this response, the Blue side also has Strike aircraft which can defend the SEADs and counteract the SAMs but are unable to attack the targets. The questions for the Blue team are: what is the value of the target and what ratio of SEADs and Strike aircraft should be deployed for the targets? Likewise, for the Red team: how valuable is the target and how many, if any, SAMs should be fired? Hamilton contends that the optimal Red strategy is to fire only for a fraction of engagement which is equal to:

(2) $$\frac{\mathrm{Value}\;\mathrm{of}\;\mathrm{Target}}{\mathrm{Value}\;\mathrm{of}\;\mathrm{Target}+\mathrm{Value}\;\mathrm{of}\;\mathrm{SEAD}\;\times Pk_{s}+\mathrm{Value}\;\mathrm{of}\;\mathrm{SAM}\;\times Pk_{A}}$$

and the optimal Blue Strategy is to assign a fraction of the planes as SEADs which is equal to:

(3) $$\frac{\mathrm{Value}\;\mathrm{of}\;\mathrm{SAM}\;\times Pk_{A}}{\mathrm{Value}\;\mathrm{of}\;\mathrm{SAM}\;\times Pk_{A}+\mathrm{Value}\;\mathrm{of}\;\mathrm{SEAD}\;\times Pk_{s}+\mathrm{Value}\;\mathrm{of}\;\mathrm{Target}}$$

where

$Pk_{s}$ is the probability of the SAMS destroying the SEADs

$Pk_{A}$ is the probability of the Strike aircraft destroying the SAMs

This formulation gives a concise prediction of the likely outcome of an engagement given every possible assignment of aircraft and missile launches. It must be noted that it is incredibly difficult in practice to accurately quantify the numerical value of different targets and resources.

Garcia et al. 2019. [74] investigate the problem of defending a maritime coastline against two enemy aircraft whose main objective is to invade the territory controlled by the defending aircraft. The defender, on the other hand, attempts to prevent this by trying to intercept both enemy aircraft in succession as far as possible from the border. This is a typical pursuit-evasion scenario and is representative of many important problems in robotics, control and defence. In this paper, Garcia et al. formulate this problem as a zero-sum differential game, where the defender/pursuer tries to successively capture the two attackers/evaders as far as possible from the defended coastline while the attackers cooperate and minimize their combined distance from the border before they are confronted. Garcia et al. then find the optimal strategies for the attackers and the defender in this one-defender two-attacker pursuit-evasion game by solving a set of nonlinear equations. The cooperative strategy discussed in this paper provides an important coordination approach for less capable (perhaps slower) agents when they are tasked to carry out a mission.

Garcia et al. 2017 [75] consider an air combat scenario where a target aircraft that is engaged by an attacking missile utilises a defending missile to defend itself as it attempts to escape the attacker by maximising the distance between itself and the attacker when the defender reaches as close at it can to the attacking missile. The game is referred to as an active target defence differential game (ATDDG). In the paper, the authors extend previous works performed on this three-party problem to develop a closed-form analytical solution for the ATDDG where the Defender missile can defeat the attacker if it enters within a capture circle with a specified radius rc > 0. Additionally, the closed-form optimal state feedback solution demonstrated in the paper is supposed to work despite the attacker employing an unknown guidance law rather than assuming it is Proportional Navigation (PN) or pursuit (P). Finally, the authors provide the set of initial conditions for the target aircraft where its survival is guaranteed if the target-defender team plays optimally despite the unknown guidance law employed by the attacking missile.

Deligiannis et al. [76] consider a competitive power allocation problem in a Multiple-input multiple-output (MIMO) radar network in the presence of multiple jammers. The main objective of the radar network is to minimize the total power emitted by the radar while achieving a specific detection criterion for each of the targets. In this problem, the radars are confronted by intelligent jammers that can observe the radar transmitted power and thereby decide their jamming power to maximize interference to the radar. Here Deligiannis et al. treat this power allocation problem as a non-cooperative game where the players are the central radar controller and the jammers and solve this using convex optimization techniques. Moreover, they provide proof for the existence and uniqueness of the Nash equilibrium in this scenario, where no player can further profit by changing its power allocation.

Similarly, He et al. [77] consider the radar counter-measure problem in a multistatic radar network, where a game-theoretic formulation of joint power allocation and beamforming is studied in the presence of a smart jammer. The goal of each radar in this network is to meet the expected detection performance of the target while minimizing its total transmit power and mitigating the potential interferences. On the other hand, the goal of the jammer is to adjust its own transmit power to interfere with the radar to protect the target from detection. First, He et al. study the power allocation game with strategy sets of each player (radar and jammer) consisting of their respective transmit powers. They then proceed to solve the corresponding optimization problems to work out the best response function for the radar and the jammer and show the existence and uniqueness of the Nash equilibria. Next, they consider the joint power allocation and beamformer design problem in the presence of jammers again as a non-cooperative game and propose a power allocation and beamforming algorithm which is shown to converge to its Nash equilibrium point.

McEneaney et al. [78] investigate the command and control problem for unmanned air vehicles (UAVs) against ground-based targets and defensive units such as Surface-to-Air Missile (SAM) systems. The motivation for this work arises from the requirement for operations planning and real-time scheduling in an unmanned air operations scenario. The problem is modelled as a stochastic game between blue players (UAVs) and red players that comprise the SAMs and ground-based targets. There can be a number of objectives for each side: for an example, a blue player may try to destroy a strategic target while minimizing damage to itself. The red players, on the other hand, may attempt to inflict maximum damage on the UAV while protecting themselves from attack by the UAVs.

The control strategies for the UAVs consist of a set of discrete variables that correspond to the specific target or SAM to attack, while that for the SAMs are to switch their radar “on” or “off”. Note that when the radar is “on”, the probability of the SAM causing damage to the blue players’ increases as does the probability of the blue players inflicting damage to the SAM. The solution to this stochastic game is obtained via dynamic programming and illustrated using some numerical examples. The main contribution of this work is the analysis of a risk-sensitive control-based approach for stochastic games under imperfect information. In particular, this approach not only handles noisy observations due to random noise but also deals with cases that include an adversarial component in the observations.

Wei et al. [79] have developed a mission decision-making system for multiple uninhabited combat aerial vehicles (UCAVs) working together. The UCAVs weapons are air-to-air missiles. In the paper, a red-UCAV team consisting of an unmanned fighter-bomber flanked by two UCAVs attempts to strike a blue-team ground target. The blue team has its own set of UCAVs that are directed to defeat the red team. The success of a given missile against its chosen threat is determined by the distance between the attacker and threats, their relative speed, and relative angles. The scenario is represented as a simultaneous normal form game with the strategies for the team corresponding with allocations of blue team entities against red-team entities and vice versa. In the paper, the payoff for a red or blue team is based upon considering the effectiveness of a given allocation, which in turn is dependant upon the relative geometry between the opposing team allocation groupings. Dempster–Shafer (D-S) theory is applied where the D-S combinatorial formula is harnessed to formulate the payoff. These payoffs, calculated for each strategy, for each team is then placed into bi-matrices, i.e., one for each team and solved using a linear programming optimisation approach. If an optimal Nash equilibrium is not present, a mixed strategy approach is applied and solved. The authors then develop some mission scenarios with differing geometries and illustrate the use of their game-theoretical allocation strategy. They use annotated diagrams of entity geometry containing red and blue teams in proximity to one another to demonstrate that the allocation strategy determined by their payoff formulation is satisfactory.

Ma et al. [80] have developed a game-theoretic approach to generate a cooperative occupancy decision-making method for multiple unmanned aerial vehicle (UAV) teams engaged against each other in a beyond-visual-range (BVR) air combat confrontation. BVR combat is enabled because of developments in missile technology enabling long-range engagements. In the paper, the team on each side first decides the occupancy positions (cubes in Cartesian space) of its UAV entities followed by selecting targets for each UAV team member to engage. The goal is for each side to obtain the greatest predominance while experiencing the smallest possible threat condition. A zero-sum simultaneous bi-matrix game is applied to analyse the problem. For a given occupancy of a UAV, height and distance predominance formulae that factor in the range and weapon minimum/maximum performance criteria are used to generate payoff values for the utility functions. As the scale of the game leads to an explosion in size (and thus strategy) as the number of occupancy cubes and UAVs for each team is increased, the authors have chosen to augment the Double Oracle (DO) algorithm that was designed to solve large scale zero-sum game problems in earlier works, by combining it with a Neighbourhood Search (NS) algorithm into a Double Oracle Neighbourhood Search (DO-NS). Through simulations, the authors illustrate that the results show the DO-NS algorithm outperforming the DO algorithm in terms of computational time and solution quality.

The work of Başpınar, Barış et al. [81] focuses on the modelling of air-to-air combat between two unmanned aerial vehicles (UAVs) using an optimization-based control and game-theoretic approach. In this work, vehicle motion is expressed in terms of specific variables and any trajectory planning for moving from one waypoint to another is solved by determining the smooth curves satisfying defined conditions in flat output space. Following determination, all variables involved to describe the smooth curve can be reverted to the original state/input space. The impact is a speed-up in the solving of any trajectory optimization through reducing the number of variables required. Game theory is then harnessed where the aerial combat between the two UAVs is modelled as a zero-sum game using a minimax approach. That is, each party tries to maximise its payoff when the opponent plays its best strategy. Here, The objective is for each UAV to get directly behind the other party and within a range threshold to satisfy onboard weapon effective range constraints.

In [81], the authors provide cost functions associated with the degree of being in tail-chase to the target based on aspect and bearing angles as well as the cost functions associated with generating a maximum score when the opponent is within some threshold of the optimum shooting range. The cost functions are multiplied together to create the total cost. The cost functions are put into a receding horizon control scheme where the trajectory planning determined through selection of the controls is performed for a given look-ahead time period where both players are utilising opposite strategies. Each player considers its opponents as reachable sets within the horizon and uses this to select its choice of controls to maximise its payoff. This process is repeated every few control steps. Unlike most other works in this area, the authors use the full set of control inputs within the performance envelope rather than a subset (e.g., turn, maintain hading, roll left at a particular angle, immelman, split S or spiral dive), and thus point to generating a more optimal solution for each player’s strategy. Two simulation scenarios are provided, with the first being the case where neither UAV starts off in air-superiority position and then exercises the receding horizon cost function optimisation to get into tail-chase with its opponent within optimum firing range. The authors show that the speed, load factor and bank-angle when applying the controls do not violate bounds during the flights and that feasible trajectories are generated. For the second simulation, the UAVs are initially in a tail-chase except not satisfying the within shooting range criterion. The opponent being chased manoeuvres to escape by applying the cost function while the chaser continues chasing. At the end of the engagement, within shooting range criteria are met and the target is directly in front but at a sub-optimal aspect, which leads to its escape. These scenarios are used to demonstrate the validity of the control strategy developed and thus provide the automatic selection of combat strategy for two unmanned aerial vehicles engaged in combat against one another.

Casbeer et al. [82], consider a scenario where an attacker missile pursuing an unmanned aerial vehicle target is engaged by two defending missiles launched from entities allied to the target which cooperate with the target. It extends from the typical three-party game scenario where there is only one single defending missile engaging the attacker cooperating with the target. The author refers to it here as an Active Target Defence Differential Game (ATDDG). Besides computing the optimal strategy for the players in the extension to ATDDG, the paper attempts to determine the degree of reduction in vulnerability of the target when it uses two defenders rather than one. A constrained optimisation problem is formulated to solve this scenario. It is shown that the target through having the choice to cooperate with either defender can more successfully escape the attacker. Additionally, the presence of two defenders enables the attacker to be more easily intercepted. When the two defender missiles are well-positioned, both can intercept the attacker.

Han et al. [83] present an Integrated Air and Missile Defence (IADS) problem where Surface-to-Air-Missile (SAM) batteries equipped with Interceptor Missiles (IM) engage the Attacker Missiles (AM) targeting cities. The problem is cast as a simplified two-party zero-sum game with perfect information and has three stages. The three stages correspond with the defender setting up their allocation of SAMs to cities, followed by the attacker allocating their missile salvo against cities and finally the defender in response allocating interceptor missiles to counter attacker missile salvo. The simplifying assumptions made in this problem are that there is only one SAM allocated near a city and only one installed per site. Additionally, no more than one interceptor is launched against each attacking missile. Additionally, only one IM can be allocated to one DM, each SAM has the same number and type of IMs, and AMs are identical and are fired in a single salvo. It is attempted to solve the tri-level game using an extensive form game tree, α−β pruning and using the Double Oracle (DO) algorithm for a six-city network that needs to be protected. The DO algorithm is a heuristic and is not guaranteed to find the Subgame-Perfect Nash-Equilibrium (SPNE). The efficiency with which the Subgame-Perfect Nash equilibrium is reached by each choice of algorithm is studied. For the game-tree approach, the conclusion made is that the size of the strategy space is determined to increase to an intractable size because of the combinatorial nature of the problem. When applying α−β pruning, compared to the DO algorithm, the paper determines that determination of the number of SAM batteries, AMs and IMs do not scale well in terms of computational time. However, the DO algorithm does fail to find the SPNE in a small number of instances. The authors prefer the DO algorithm despite this, as it is shown to not violate monotonicity (increase in payoff) and the solution quality trends (non-exponential increase in computational time) even when increasing the size of the problem from 6 cities to 55 cities.

### 3.4. Papers Dealing with Cyber Warfare

Papers that deal with applications of game theory in Cyber Warfare, as distinct from Cyber Security, are few. Significantly among them, Keith et al. [84] consider a multi-domain (cyber combined with air-defence) defence security game problem. Two players are engaging each other in a zero-sum extensive form game, a defender, representing an integrated air-defence system (IADS) equipped with cyber warfare protection, and an attacker, capable of unleashing air-to-ground threats (missiles, bombs) as well cyber-attacks (against the IADS network). Here, the payoff has been selected as the expected loss of life. The defender wants to minimise this while the attacker wants to maximise it. The cyber security game problem to protect the IADS is nested within the physical security game problem. The actions of the players correspond with allocations to activate IADS/cyber security response nodes corresponding with population centres for the defender, and allocations to attack IADS/associated cyber-security nodes by the attacker. The realism of the game is increased through provisioning in imperfect information; that is, the defender and attacker are not fully aware of the level of vulnerability of nodes. Additionally, the defender is only able to sense cyber attacks on nodes probabilistically which implies that its allocation of cyber defence teams to particular IADS is only effective probabilistically. For the attacker, it can also determine the effectiveness of its cyber-attacks following physically attacking a node. This work is primed to advance the security game literature by introducing the integrated domain, multiple periods for agent actions and enabling continuously mixed form strategies by the players. The author considers it the first work where Monte Carlo (MC), and discounted and robust Counterfactual Regret Minimisation (CRM)-based approaches have been compared in security games. Initially, for a small-scale version of the problem, the Nash equilibrium (NE) in the form of a sequence-form linear program is determined for the defender. Then, the problem is gradually scaled up to include additional population centres to be defended up to an upper limit. Here, an approximate CRM algorithm is introduced to reduce computation time while preserving the optimality of a particular strategy as much as possible. When the scale is further increased, a discounted CRM is introduced which further reduces computation time.

The parameter space of the problem and algorithm are explored to select the best choice of tuning parameters and extract the best performance from the algorithms. The rationality of the players is made limited by introducing bounded rationality so that they do not necessarily make the optimal responses. They can only manage approximate robust best response moves. A robust best response for a player is defined as the compromise between the completely conservative NE strategy and the completely aggressive best response strategy. It introduces weaknesses in players’ strategies. With respect to a player, the capability of their strategy to capitalise over an opponent’s strategy is referred to as exploitation. Conversely, the vulnerability of their strategy with respect to an opponent is referred to as exploitability. When running all the different algorithms introduced, the results show that the Nash equilibrium solution is the safest strategy since the best moves are being played which are not exploitable, however, it does not produce the highest utility for a player. The performance charts show that the robust linear program generates the highest mean utility and highest exploitation-to-exploitability ratio while also consuming the maximum computational time. The Data biased CFR is seen to offer the best trade-off by offering a high mean utility, an exploitation-to-exploitability ratio in favour of exploitation while running in the lowest computational time.

### 3.5. Papers Dealing with Space Warfare

In the domain of space warfare, human resources and risks are much less prevalent, and so the focus is more on network strength and interaction between independent autonomous agents, connected or otherwise. Ultimately, warfare in these aspects will operate at a pace and in dimensions far beyond human cognitive capacity. Since the rapidity and complexity of decisions within engagements will almost certainly outscale military personnel’s understanding, the game theory will take the place of decision-makers as part of the overall software and control system, and imbue future technology to consider human/social factors when making calculations. With a greater focus on connectivity and networking, the key to success in these areas relies on effective communication channels and a shared goal across the system. In this nascent area of research, papers that apply game theory are often concerned with satellite networks.

Zhong et al. [85] set the ambitious goal of optimising bandwidth allocation and transmission power across a satellite network. They base their research on bargaining game theory and have to achieve compromise across interference constraints, Quality of Service requirements, channel conditions, and transmission and reception capabilities for satellites at every point in the network. Interference constraints and bandwidth limitations are the surpluses that need to be negotiated in the bargaining game, with each satellite using different strategies to improve its utility/share of resources. This quickly escalates in complexity, with the most important takeaway from the model being the mapping of a problem to the cooperative bargaining game framework.

Similarly, Qiao and Zhao [86] detail some key issues with the finite energy availability for the nodes in satellite networks. Their paper offers a solution through a game-theoretical model of a routing algorithm and uses it to find an equilibrium solution to the uneven network flow. The model locates certain network hot spots, which are reserving a lot of energy and takes measures to evenly distribute the resources. This is another case of a bargaining/cooperative game across multiple players in a network.

### 3.6. Papers Dealing with Target Tracking

Since target tracking is an established research area, we found several papers applying game theory tracking problems. Most of these have overlapping warfare domains and do not put too much emphasis on demonstrating applicability in a particular domain. For example, Gu et al. [87] study the problem of tracking a moving target using a sensor network comprising of sensors capable of providing some position-related target measurements. Each sensor node has a sensor to observe the target and a processor to estimate its state. While there is some communication available among sensors, this ability is limited in the sense that each sensor node can only communicate with its neighbours. The problem is further compounded by the fact that the target is an intelligent agent capable of minimizing its detectability by the adversary and thereby has the potential to increase the tracking error of the tracking agent. Gu et al. [87] solve this problem within the framework of a zero-sum game, and by minimizing the tracking agent’s estimation error, a robust minimax filter is developed. Moreover, to handle the limited communication capability of the sensor nodes, they propose a distributed version of this filter for which each node only requires information in the form of current measurement and estimated state from its immediate neighbours. They then demonstrate the performance of their algorithm on a simulated scenario with an intelligent target and show that while the standard Kalman filter errors diverge, the minimax filter which takes into account the adversary’s noise, can significantly outperform the Kalman filter.

Qilong et al. [88] similarly address the issue of tracking an intelligent target, but they model a scenario where the tracking players are also in pursuit, and the focus is on protecting the target. Additionally, the target can fire a defensive missile at the attacker/tracker. The attacker has a line of sight of both the target and the defensive missile. The target plans to allow the tracker to slowly close the distance between itself and the target, all the while manoeuvring to develop an understanding of how the attacker reacts. When the attacker is close to collision, the defensive missile is released. The target and the missile then communicate, use the knowledge of the attacker’s movement patterns, and adhere to an optimal linear guidance law to destroy the attacker. This was modelled as a zero-sum competitive game between the attacker, the target, and the defensive missile. However, the paper also focuses on the cooperative game played between the target and the defensive missile, which is a non-zero-sum game. For them, the payoff is calculated by minimised miss distance (which ideally equals zero—a collision with the attacker), as well as the control effort required to guide the defensive missile.

Faruqi [89] discusses the general problem of applying differential game theory to missile guidance. They state that missile trajectory follows Proportional Navigation (PN), a guidance law typically used by homing missiles. The performance of these systems is measured by a Linear System Quadratic Performance Index (LQPI). With respect to differential game theory, they model the missile guidance problem by representing the missile navigation and trajectory with a set of differential equations. The general form of this problem is

(4) $${\underline{\dot{x}}}_{ij}=F{\underline{x}}_{ij}\left(t\right)+G{\underline{u}}_{i}\left(t\right)-G{\underline{u}}_{j}\left(t\right)$$

and

(5) $$J\left(\ldots \right)=\frac{1}{2}{\underline{x}}_{ij}^{T}\left(t_{f}\right)S{\underline{x}}_{ij}\left(t_{f}\right)+\frac{1}{2}\int_{t_{0}}t_{f}\left[{\underline{x}}_{ij}^{T}QG{\underline{u}}_{i}+{\underline{u}}_{i}^{T}R_{i}{\underline{u}}_{i}+{\underline{u}}_{j}^{T}R_{j}{\underline{u}}_{j}\right]$$

where

${\underline{x}}_{ij}\left(t\right)=x_{i}\left(t\right)-x_{j}\left(t\right)$: is the relative state of player *i* w.r.t player *j*

${\underline{u}}_{i}\left(t\right)$: is the player *i* input

${\underline{u}}_{j}\left(t\right)$: is the player *j* input

F): is the state coefficient matrix

*G*: is the player input coefficient matrix

Q): is the Performance Index (PI) weightings matrix for current relative states

S): is the PI weightings matrix for final relative states

$R_{i},R_{j}$: PI weightings matrices on inputs

Faruqi mainly focuses on two-player and three-player games, while the utility functions are modelled based on the relative distance vectors between missiles and targets. Faruqi shows that game theory can be effectively used in missile guidance tasks involving PNs in modern missiles.

**Figure 4:**
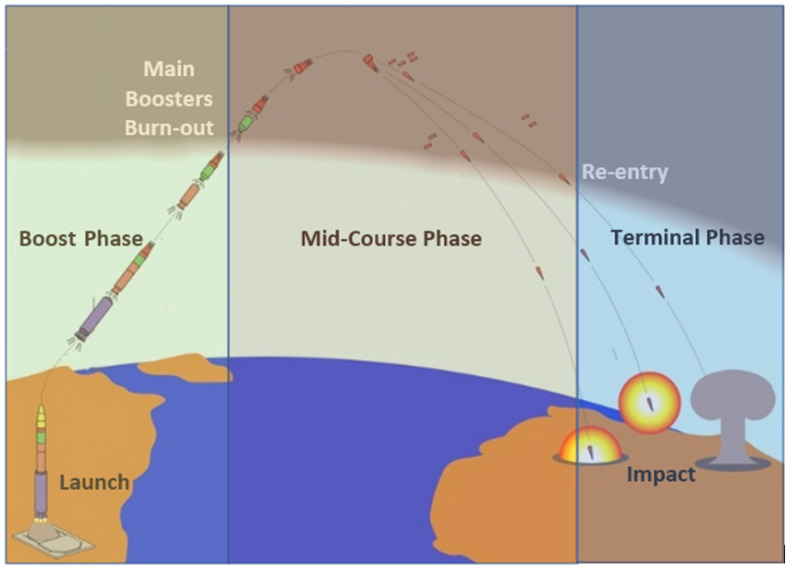
Flight path of Theater Ballistic Missiles.

Evers [90] on the other hand analyses defence against Theater Ballistic Missiles (TBMs) using game theory. The proliferation of ballistic missiles and nuclear technology has important consequences for military conflict, where the cost of failure can lead to the destruction of entire cities. It can be hard to pinpoint their launch as they have a big range, are very powerful, though their payload can vary considerably. In combating this threat, the defending nation does have the advantage that there is usually a long flight trajectory, typically split into three phases, during which the TBM can be intercepted. The Boost phase marks launch and the majority of the TBM ascent. The end of the boost phase is marked by the burn-out after which the TBM enters its midcourse phase. This phase is the longest phase of the flight and affords the best opportunity for defenders to intercept the TBM. After the midcourse phase, the TBM enters its terminal phase from re-entry into the atmosphere. This is the last opportunity for defenders to intercept the missile. The flight path is illustrated in Figure 4 below.

The missile travels a long distance over a reasonably extended flight time. However, from its physical geographical location, a defending military force or nation can only apply its resources to defend against it in the termination phase of flight, where the risk is much higher and the cost of failure is at its greatest. For this reason, Evers proposes a cooperative strategy, where the defending nation forms alliances with nations around itself so that they too can attempt to intercept the TBM in its earlier phases as it travels to the impact location. Therefore, the game is divided into two smaller games: the first being a cooperative multiplayer game devising a set of strategies for the coalition of nations to utilise throughout a TBM’s flight path, and the second is the bargaining and cooperation game between the defending nations and potential allies.

The basis of the cooperative game to shoot down the TBM is a strategy called ‘shoot-look-shoot.’ It relies on a set of *N* nations attacking the target using a set of strategies—their interceptor missiles—*M* each of which has its own Probability of Interception $P_{i}$. As the TBM flies, each nation *n* in *N* will fire its missile(s) $m_{n}$ to intercept the TBM, then look to see if it has successfully neutralised the threat. If it has failed, the next nation’s missile(s) $m_{n+1}$ will be fired. The game’s problem is then reduced to optimising the probability of interception of the whole set of strategies, such that it has a feasible likelihood of stopping the TBM. Game theory is useful here because the principles of cooperative game theory provide a strong mathematical framework by which an equilibrium solution can be reached for the set of cooperating nations.

The second game described by Evers is based on negotiating with other nations to form an alliance. For these other nations, participation in this game is a risk because it makes them another potential target for the attacking force. To solve this game, the defending nation must accurately evaluate the interceptor cost savings, that is, how much there is to gain by preventing the impact of the TBM. With these savings becoming the surplus that cooperating nations can share in, potential allies then negotiate over how those savings will be shared, in proportion to what interception resources they have to offer.

Shinar and Shima [91] continue the research of both pursuit-evasion games and ballistic missile defence with a zero-sum game of a highly manoeuvrable ballistic missile avoiding an interceptor missile. More specifically, it ties in an imperfect information element to the game, where the ballistic missile knows it is being attacked by anti-missile missiles, but knows little about their trajectory or launching locations. In this game, the two players are the ballistic missile and the interceptor. If the ballistic missile uses a pure strategy, it will likely be hit because it either (a) cannot react quickly enough to an opponent it has little information about or (b) will move predictably and allow for a straightforward trajectory towards collision. Therefore, the best solution to the game for the ballistic missile is in mixed strategies.

The mixed strategy will incorporate stochasticity in its flight pattern, assigning a probability distribution over a set of pure strategies. These pure strategies will be based on essential navigational heuristics, which will likely be known or easily discovered by the interceptor. By applying a small number of rapid and random switches in strategy, the ballistic missile can maximise its potential for avoiding interception, and force the complexity of timing calculation back onto the interceptor.

Bogdanovic et al. [92] investigate a target selection problem for multi-target tracking using a game-theoretic perspective. This is an important problem in a multi-function radar network as it needs to perform multiple functions such as volume surveillance and fire control simultaneously while effectively managing the available radar resources to achieve specified objectives. Thus, in effect, they tackle a radar resource management problem in [92] and use non-cooperative game-theoretic approaches to find optimal solutions for this problem. They formulate the problem in a framework where each radar is considered to be autonomous; there is no central control engine informing the radars of their optimal strategies nor is there any communication among the radars. First, they consider a case where all radars share common interests with respect to the targets and for this problem, they propose a distributed algorithm based on the best response dynamics to find the Nash equilibria points. This problem is then extended to a more realistic case of heterogeneous interests among radars and partial target observability. For this case, they employ the solution concept of correlated equilibria and propose an efficient distributed algorithm based on regret matching which is shown to achieve comparable performance to the more computationally intensive centralized approach.

Finally, Parras et al. [93] examine a pursuit-evasion game, involving anti-jamming strategies for Unmanned Aerial Vehicles (UAVs). The game operates within a continuous time frame and is therefore dynamic, being solved with the help of differential Game Theory. In somewhat of a culmination of the aforementioned work, it combines elements of communication optimisation, sensor evasion and navigation. Given that UAVs require strong communication for control and the relaying of information, this dependency makes UAVs incredibly susceptible to jamming attacks. There are multiple strategies to both jam and anti-jam these communications, and this can be considered a zero-sum game where the UAV must attempt to optimise its communication capacity. There is usually uncertainty about the positioning and movement of the jamming agent, so the game is an imperfect information differential game. The most important payoff for the UAV is avoiding losing communication to jamming, and it can do this by manoeuvring to make approximations for the distance of jamming agents and thus avoiding them.

### 3.7. Papers Dealing with National Security

The key components of homeland security addressed by game theory are cyber security, modelling terrorism threats and defence contracts. With many applications in computer science the [94,95,96], game theory fits well into cyber security problems. Game theory combines the strict mathematical rigour of computer science, with more psychological and philosophical elements like attacker incentives and mindset, as well as human vulnerabilities in cyber security. Terrorism modelling similarly benefits from the psychological flavour of game theory, since so much of the impact of terrorist activity is not easily quantifiable, including social, economic and other spheres affected by terrorist threats, all of which are modelled in a game-theoretic setting. Finally, game theory is suitable for a topic such as contracting and subcontracting because it captures interactions between selfish individuals effectively [1,2], and this has been used to model the behaviour of defence contractors.

The paper by Litti [97] provides a brief summary of how traditional network security heuristics can be updated with more precision, and how game theory can help network security engineers design strategies to properly predict, mitigate, and handle threatened networks. He develops a qualitative method for valuing the potential risks and costs of attacks on networks. While a fairly short paper, it does provide some cyber security situational examples of game theory in practice. For example, he models a two-player zero-sum game to represent an attacker and a security system. The nodes have their own interdependence, vulnerabilities and security assets, but cooperate to minimise the attacker’s potential to compromise the system.

Jhawar et al. [98] offer a more specific approach of game theory, namely, Attack-Defence Trees (ADTs), to model scenarios involving cyber security threats. Here ADTs are used to map potential attack and defence scenarios on a system that is equipped with automatic defence protocols. The system needs to be comprehensive in addressing all possible vulnerabilities, as well as generating responses that adapt to the aggressively evolving situation of cyber security attacks. Currently, ADTs only provide upfront system analyses. Having a reactive strategy for cyber security is important because Attackers constantly change their attack strategies for offence, and so the timing of real-time responses can make the difference between a successful and a failed defence of a system. In Jhawar et al. [98] they model a simple game of Attacker and Defender—a hacker and a security network administrator. The hacker attempts to breach the integrity of the system, and for each move they make, the administrator will have devised a reactive strategy based on the attacker’s attempt. The greatest utility of this method comes from the ability to convert long extensive form games into a graphical layout for easier understanding and communication.

Gonzalez [99] clearly outlines a standard two-player competitive game of attacker and defender, and then utilises Instance-Based Learning Theory and Behavioural game theory. The former compiles cognitive information into a representation known as an instance. Each instance has a three-part structure of situation, decision and utility—a standard game. However, it is the interaction between instances that is critical to this approach. Instance-Based Learning Theory uses the learning from the outcome of each instance to feedback into the situation of the next instance, hopefully leading to better decisions in later iterations. This is notably similar to the reinforcement learning technique in machine learning. On the other hand, Behavioural game theory involves devising a strategy where we assess a variety of factors, to make more precise long term evaluations of targets and resources so that utility scores more closely reflect real-life value. Once again, game theory facilitates access to social information in cyber security applications and assesses how that will affect the behaviour of both players in the game. Other critical factors include motivational factors of the players, completeness of information for each player, and technological constraints and inefficiencies between player and technology. Gonzalez stresses that the importance of accommodating these factors in any cyber security model will help make more realistic and useful policies for cyber defence.

One common use of cyber security is in the prevention of terrorism. Hausken et al. [100] cover both terrorism and natural disaster modelling with some guiding game theory principles. Terrorism and Natural Disasters are addressed by defending with anti-terrorism, anti-disaster, and anti-all-hazard investments. Making projections about the likelihoods of each of these occurrences, defenders must make strategic decisions towards the amount of investment dedicated to each defence. Costs to be considered in the utility function of each of these situations include the intelligence of terrorists or the randomness/ environmental control of natural disasters; the intensity of an attack/ disaster, and differences in evaluation of target value between a terrorist and defender. The game-theoretic approach used in this analysis captures the defender’s effort in combating each threat. Depending on the likelihood of each event, combined with the costs of each defensive system, defenders can derive an optimal division of funds.

Kanturska et al. [101] present a rigorous inspection of how transport network reliability can be assessed using game theory when attack probability on different locations is unknown. The approach favoured using a minimax algorithm to distribute risk across multiple paths as long as the travel costs are small relative to potential losses incurred by an attack. This would be useful in assessing the potential risk associated with safely escorting a VIP through a city. Game theory is helpful in this situation because it can analyse network reliability when the attack probabilities are unknown.

Bier [102] presents useful game theory-based suggestions for policy insights and investment decisions, insurance policy premiums and more. Her work discusses the weakest link model: a strategy focusing all resources on preventing the worst utility scenario. This is generally not ideal in practice, and she suggests instead to consider hedging those investments with a variety of defence strategies for different potential targets. The paper considers terrorist/defender games, and how security investments can change the landscape of attacker-defender interactions for the whole community. This is mainly done through its own scoping study, with one of the key takeaways being that terrorism mitigation systems can benefit from game theory because it adds an extra layer of consideration of the terrorists’ response to any defence mechanisms. Hence, game theory, combined with the holistic approach of risk and reliability analysis over all systems, can provide a more comprehensive assessment of all the potential risks and vulnerabilities in counter-terrorism strategies.

Cioaca [103] investigates a similar question to Bier et al. [65] as mentioned earlier but specifically focuses on aviation security. The problem is summarised by targeting both the cost of security measures for airports and the cost of maintaining a stable and resilient system of defence. Key strategies are: preventing the attack or threat entirely (either by removing all access to the target location or restricting airlines permissions if they fail to adhere to imposed guidelines); managing the temporal dimension of the attack (the length of the attack and the subsequent time to recover from it); understanding all direct and indirect losses (both casualties and related damages like contamination or infection, compromised secondary security measures, or reputational/signal ramifications); and the costs of mitigation, response and recovery.

The model is built around several factors and parameters. The first and most critical is human losses and material damages $C(H,Db_{t})$. *H* refers to human losses, *D* refers to material damages and $b_{t}$ refers to the budget allocated to the associated security system involved. The most obvious and direct casualties of an attack, these two losses are highly negative payoffs in such an attack and are often greater than any cost to prevent them. Human losses *H* are hard to numerically quantify, and therefore when making proper assessments about resource division, understanding how to minimise human losses across different groups of humans and in different dimensions is one of the most difficult aspects of this problem. Material damages *D* can be monetarily quantified, but often the run-on effects of such damages are where the significant losses are incurred. These losses can lead to total infrastructure shutdowns, ceasing operation of the facility, loss of jobs of workers and even potentially the slow decline and shutdown of the facility altogether. The second major factor in this game is the budget allocated to security systems $b_{t}$. Organisational and managing bodies will only have a certain amount of resources allocated to a security system *T*. The next factor is the number *s* of security system components as this will be how the budget is composed. Each of these components are partitioned into one of *n* separate system sub-components. These components are divided amongst a number of targets $t_{ik}$, and each one of these targets is assigned a probability of being attacked $p_{a_{j}}^{t_{k}}\left(b_{t_{k}}\right)$ and a value $w_{t_{ik}}$. This can be formally expressed as:

(6) $$\min C\left(H,Db_{t}\right)=\sum_{j=1}^{l}\sum_{i=1}^{n}\sum_{k=1}^{s}p_{a_{j}}^{t_{ik}}\cdot p_{v_{j}}^{t_{ik}}\cdot w_{t_{ik}}$$

For any system of resource division, Ciaoca advocates establishing dimensions of measuring system resilience. This is divided into static resilience, the efficient allocation of resources; dynamic resilience, the recovery speed of the system after the shock, including long term investment in-flows. These two forms of resilience signify the strength of a system both before, during, and after an attack. With respect to game theory, Ciaoca’s study defines a game clearly and incorporates a myriad of complex and interconnected parameters to outline an effective and calculable game model.

The final paper we discuss on national security is by Gardener and Moffat [104]. This paper covers the notion of developing a strategy to assess defence contractors and their potential performance/ ability to meet contractual obligations. In game-theoretic parlance, this problem can be expressed in terms of cooperation and defection. Gardener and Moffat suggest quantitative methods through which defence departments can more rigorously assess contracts and bidding scenarios, and therefore wisely select contractors, and protect their budget. Gardener and Moffat further the understanding of change requirements in project management at different bidding stages of defence acquisition projects. The factor they focus on is the *conspiracy of optimism*, whereby projects spiral out of control—past budget limits and necessary deadlines—due to irrational expectations of project progress. Often this ’conspiracy’ is a drive towards making short term gains, and in fact leads to overall losses. The bidding game that is played becomes less about the success of the project, and more about profit capitalisation, and can further degenerate into a two-player game where the relevant defence department is against the industry of contractors as a whole.

### 3.8. Papers Dealing with Other/Mixed Warfare

Some papers use game theory in defence settings, but cannot be easily classified into any of the types mentioned above, or are concerned with mixed warfare. For example, Zhang and Meherjerdi [105] investigate how groups of multiple unmanned vehicles can be used and controlled using game-theoretic methods in different communication frameworks. Dividing a mission for a single unmanned vehicle amongst multiple unmanned vehicles yields more effective task allocation and performance. The separation of labour away from one powerful single vehicle to several smaller vehicles provides flexibility, adaptability and improved fault tolerance. The uses of such a network are surveillance, exploration, satellite clustering, combining Unmanned Underwater Vehicles (UUVs) and submarines, planes and Unmanned Aerial Vehicles (UAVs), and cooperative robot reconnaissance. As evident from this list, the strategy is incredibly powerful because of its ability to combine resources across multiple domains. To be able to operate a cohesive unit of resources across multiple unmanned systems for combat or exploration brings an unprecedented level of information and control which would accelerate military performance tremendously.

Similarly, it could be noted that search is a ‘hide and seek’ game with a long history in military applications [106,107,108,109,110,111,112,113,114,115]. The theory was pioneered by Koopman [106] primarily in the military context (search for an escaping target), followed by developments by Stone et al. [107]. The applications include submarine hunting, mine detection, rescue operations, the risk for the first responders, and localization of a hazardous source [106,107,108,109,110,111,112,113,114,115]. This framework provides the optimal *a priori* search plan for a given detection model, target motion and the cost of the search. The cost of the search may include time of the search, probability of escape (for a target), exposure risk (for a searcher), information entropy, or situation awareness (map of probability of target location). The searcher can be a moving platform (UAV, UUV, patrolling boats, helicopters, robots, people) and the targets can be static, movable, blind, silent, or emitting. In this context the simultaneous localisation and mapping (SLAM) algorithms are often used [116]. The new direction of research in this niche (inspired by some biological applications) employs the ideas of infotaxis [117], or real-time control of the searchers’ movement based on information (entropy) gain extracted from the environment (sporadic measurements, forbidden areas, communication between searchers). Principles of game theory could be applied in such contexts which can be modelled as ‘hide and seek’ games.

## 4. Classification and Impact

### 4.1. Classification of Papers

In the previous section, it would have been noticeable that many papers have applicability in more than one domain, and use myriad types of games and model a range of players. It is therefore imperative to classify the reviewed papers in a principled manner. To do so, we use the classification scheme already introduced in Table 1 in Section 2.

In particular, the reviewed papers could be classified based on (1) the domain or type of warfare (2) the type of game or games used in the paper, and (3) the nature of players modelled in the paper. The domain can be broadly classified into Traditional (T) or Modern (M), and more specifically into Land warfare, Naval warfare, Aerial warfare, Cyberwarfare and Space Warfare. The type of games used can also have a complex classification, based on whether the games were Non-cooperative or cooperative, sequential or simultaneous, discrete or continuous, zero-sum or non-zero-sum. Finally, the games can be two-player, three-player, or multi-player (more-than-three player) games. All of this is succinctly captured in Table 1.

In Table 2, we provide a self-explanatory, elaborate classification of all the reviewed papers based on the above-mentioned classification scheme.

### 4.2. Impact-Related Metrics of the Papers Reviewed

We now consider the question of which of these papers have attracted the most interest in the research community or resulted in follow-up or related work. One generally used metric to measure such impact is citation count, though obviously this metric is biased towards earlier papers. Nonetheless, in Table 3 we present the Google Scholar citation count of the 29 papers considered. It is self-evident to the reader which papers have attracted the most citations, and we will not further comment upon it. We stress, however, that citation count is not the only measure of impact, nor it is necessarily the most effective to gauge the impact of a paper in the research field. However, it is a readily available measure that conveys useful information.

Table 3 also shows the country of origin for each paper considered, being defined as the country that appears in the corresponding author’s first affiliation. It can be seen that the papers considered were written by researchers from the United States, the United Kingdom, Australia, China, Netherlands, Canada, Israel, India, Germany, Spain and Romania. It appears that the primary interest in applying game theory in defence science exists in the US, Europe (particularly Western Europe) and China, while it is acknowledged that there may be several papers written in languages other than English which we did not consider.

To understand whether there was sufficient cross-pollinating of ideas across the different domains described in the above classification, we considered how many papers among the 29 reviewed papers cited others from the same set. The citations, according to Google Scholar, are presented in Table 4. Surprisingly, there was no paper that was cited more than twice by other papers which are reviewed, and most papers are not cited by other papers in the set at all. This is despite the fact that the overall citation count of the papers in the set is healthy—the reviewed papers are cited 34.97 times on average according to Table 3, and several papers are cited more than 50 times. Most of these citations, however, seem to come from papers concerned with defence science and technology which use various methods and tools to solve similar problems, and it is obvious that there is little cross-pollination between researchers who use game theory in defence applications. Therefore, quite apart from ‘gaps’ in the literature which we present below, which indicate potential research opportunities, it should also be emphasized that awareness of similar works in the field should increase, and this will likely result in ideas generated in a particular domain being re-used in other domains and other applications related to defence.

## 5. Opportunities for Further Research

The reviewed papers have shown that game theory can provide a unifying framework to analyse the decision-making behaviours of agents in defence contexts. In this section, we briefly discuss a range of potential defence scenarios where game theory has hitherto not been applied but would make a useful contribution if applied in future.

A recent investigation by Defence Advanced Research Projects Agency (DARPA) into ‘Mosaic warfare’ [118] is an example of such a potential future application of game theory. The idea is mentioned in Zhang [105] in the context of operating multiple unmanned vehicles, and proposes having a lot of smaller cost-efficient resources interconnected in a ’mosaic’ network, such that if several units are destroyed, the overall integrity of the network still remains, much like how a mosaic retains its image even if a few tiles are removed. The goal is that such a vast array of resources with different capabilities will be able to overwhelm the enemy with its completeness and complexity. It utilises principles of concurrency to address the intricacy of the connections in systems of millions of sensors and actuators. These systems in turn must handle inter-system communication. If successfully implemented, such a system of systems can provide military strategists with an overwhelmingly powerful network of weaponry and resources, which can defeat opponents with the sheer scale and complexity of their dynamics. This method of combining different parts of the arsenal maximises the benefits of each, and reintroduces a focus on expendability of resources, rather than focussing on a few pieces of high value weaponry. This in turn builds resilience and adaptability into the strategy, a shift away from heavyweight, single focus attack methods. Since there are a high number of lower cost resources which need to cooperate for the best outcome, this scenario could be modelled as a multi-player co-operative game at one level, while the strife with the opponent(s) can be modelled as a multi-player non-cooperative game. It can be noted that the concept of ‘Mosaic warfare’ is essentially similar to the more general concept of agent-based modelling, which has already been used in several diverse contexts, ranging from Ageless Aerospace Vehicle design [119] to modelling of infectious disease dynamics [120], and game theory has already been used successfully in some of these contexts [120,121].

Another area, within the context of naval warfare, where game theory could be fruitfully applied is in naval susceptibility. In analysing naval susceptibility, naval vessels factor in their environment, movement patterns and potential adversary sensors to calculate their risk of detection when moving as a covertly [122]. Such an application has overlaps between commonly studied tracking problems in defence science, as explained in Gu [87], which describes tracking using sensor networks. Such a scenario, as elaborated before, could be modelled as a two-player non-cooperative differential game, with detection being the main payoff parameter for each player.

Indeed, ground-based tracking problems could benefit from the application of game theory as well, and papers in this area so far have been few. Ground-based tracking problems may appear in both ground-based military applications (classified here as land warfare), as well as domestic security and anti-terrorism applications (classified here as national security applications), where the ability of security agencies to track individuals’ movements throughout a society—including their locations, their social network and their motivations—is a crucial capability [123]. The latter scenario could be modelled as a two-player game of pursuit and evasion, or perhaps just pursuit and reconnaissance with the aim of not revealing the pursuit to the target, while the target would try to identify pursuit. The amount of predictive information gained from covert tracking would be the payoff in this situation.

Modelling cyber warfare is another area where game theory could be applicable, and again, as noted in the relevant section earlier, there have been few papers addressing this niche, other than papers coming primarily from the computer science domain where the focus has been primarily on cyber security. Kim et al. [124] describe cyberwarfare scenarios that are integral to all military operations and highlight the critical role played by new technological paradigms such as the Internet of Things (IoT) and Brain Computer Interfaces. Defence experts increasingly need to predict and preempt cyberwarfare strategies of hostile players. Modelling decision-making scenarios involving Cyberwarfare scenarios with novel technological interfaces is an area where game theory can play a vital role.

As mentioned above, it may also be considered a ‘gap’ in the presented literature that there seems to be little cross-pollination, exchange of ideas, or even awareness of other works which are similar, in this niche, according to Table 4. Therefore, increased collaboration among researchers who use game theory in defence applications is desirable and will result in the reuse of game-theoretic methods across multiple domains of warfare.

## 6. Discussion

It is pertinent here to discuss how a review such as this adds value to the field, beyond summarising the state-of-art and identifying gaps in the literature. It was observed that citations from one paper to another within the selected set of papers are rare. While it is hard to pinpoint the reasons for this, it could be observed that most studies focus on specific domains of warfare, such as land, naval, or aerial warfare, and try to address specific problems in a particular domain of warfare. Therefore, a paper that focusses on a different domain of warfare was perceived not necessarily as another paper that applies game theory in a defence context, but as a paper that belongs to a different domain of warfare, and therefore was not looked into. Yet it is clear that such an approach can result in missed opportunities as there is often no consideration about where else the same set of game-theoretic tools could be applied similarly. A review paper such as this will go some way to rectify this problem. Moreover, it can be observed that payoff functions are often rigidly and narrowly defined, based on what has been traditionally thought of as important parameters in a particular type of warfare. For example, land warfare focuses on minimising casualties, while national security applications focus on boosting public confidence. Yet, in most types of warfare, there are a range of factors that contribute to the ultimate payoff, ranging from casualties and loss of military assets to public confidence, indirect economic costs, opportunity costs, costs to the allies, and political and strategic considerations. This review paper is likely to stimulate the modelling of more holistic payoff functions in each domain of warfare where game theory is used, by presenting a broad overview of payoff structures across several warfare domains. Furthermore, in a generic sense, this review will help to highlight defence-related decision making as a methodical and rational process that is amenable to structured analysis, as opposed to being an intuitive process as it used to be perceived in some sections of the defence community [125]. At the same time, the presented analysis will help avoid micromanagement on one hand and impulsive decision making on the other hand [126], instead of encouraging quantitative decision making processes in defence applications.

In particular, beyond the operational and tactical decision-making processes, the presented review has managerial and social implications.

### 6.1. Managerial Implications

The application of game theory can be very useful to the defence forces of a country, not only in tactical and operational matters but in strategic management of the defence assets in peacetime as well. For example, the strategic placement of military resources, such as battleships, submarines, and fighter jets, for purposes of deterrence as well as operational readiness, can be treated as a cooperative game or equivalently an optimisation problem which can be solved by linear programming or nonlinear programming. Similarly, decisions regarding the placement and building of strategic military facilities, such as bases, airstrips, harbours, or even roads and railways [127,128] can be aided by modelling the related scenarios using cooperative game theory. Another managerial decision-making process where game theory can be applied is the management of reserve military personnel, including when to call up reserves. Therefore, game theory is useful not only to military personnel who make operational decisions but also to civilian managers and politicians who have to make defence-related decisions, including in peacetime, which may have long term consequences.

### 6.2. Social Implications

The social benefits from applying game theory in defence scenarios arise primarily from the viewpoint of national security. Public perception of homeland security is an important part of defence considerations and has considerable influence on defence spending [129]. Decision-makers from defence and law enforcement need to consider not only the actual risks and threats, but also perceived risks, and factors that are influenced by it, such as insurance costs, the effect on tourism, the ratings of credit rating agencies, the willingness of investors to invest in a country, the actual and perceived cost of security measures etc, in making decisions about defence spending. Game theory can be a very useful tool in modelling such a complex set of factors and parameters, and the resulting overall payoffs in different scenarios. Conversely, such decisions, once made, obviously affect national security and in turn influence public confidence and perception of national security. Therefore, perception of risks and spending on national security influence each other [130,131] and the toolset that game theory offers is very useful to model such a complex feedback loop. Obviously, the public mood and perception of events is important in wartime scenarios as well, where decisions made in all domains of warfare will influence public perception, which may, in turn, affect the trajectory of the conflict. Therefore, the application of game theory in defence scenarios has clear social implications.

It is also important to note that this review adds fresh insights which can be useful in understanding command and control warfare. One such insight is that cooperation and hostile competition between intelligent agents are not so fundamentally different as they appear to be at first sight: indeed, in a sense, they can both be represented by the same framework (game theory), and both involve a number of intelligent players, strategies, and payoffs. The difference is that hostile competition is represented by Non-cooperative game theory, where one player’s payoff increase often results in another player losing out (a zero-sum game is a special case of this). Cooperation on the other hand is represented by cooperative game theory, where we model coalitions and sometimes discuss the concept of ‘public good’. Such an insight is especially useful when a hostile player can potentially turn into an ally or vice versa. Another insight is that the primary difficulty in modelling defence scenarios as games arises not from identifying the possible strategies or the players, but from quantifying the payoffs. Quite often, the papers that we have reviewed make several assumptions, simplifications, and estimations in quantifying the payoffs, and it is possible to envisage that the accumulated errors introduced by these processes may have critically altered the outcome of the game, and thus rendered the modelling ineffective. Therefore, the primary challenge many papers have faced in applying game theory is to model the payoffs accurately or sensibly. Such insights cannot be gained unless a broad review of several defence applications of game theory is compiled, as we have done here, and these insights are important in shaping the directions of future research in the field.

## 7. Conclusions

Game theory has proven itself as a versatile and powerful tool for obtaining vital insights into the decision making processes of agents and players in a number of fields. In this review paper, we elaborated several scenarios in which game theory could be applied in defence science and technology, and presented a succinct review of existing research in this direction. We introduced an extensive classification for the twenty-nine reviewed papers, based on the kind of warfare studied, type of games used, and the nature of players. Based on the observations made, we identified gaps in the literature where game theory has not been applied extensively so far but has great potential to be applied fruitfully in future; and we discussed the potential directions in which defence applications of game theory could expand in the future.

The domain-based classification was the primary mode of classification that was employed, and in this context, we grouped the reviewed papers into the land, air, sea, cyber and space domains. We also considered papers primarily concerned with tracking and national security. For each paper considered, the number and roles of players and game types were defined, and where possible, strategies and payoff functions were discussed. The goal of this exercise was to identify the most commonly analysed domains as well as frequently used game types, and use this knowledge to identify gaps in the literature and cross-pollinate ideas across various domains and types of warfare within the defence context.

It is hoped that this review will result in several positive outcomes. We have identified gaps in the literature and pointed out that the toolsets offered by game theory are not fully harnessed in analysing certain modes of warfare. For example, we pointed out, there are relatively few papers about naval warfare which use game-theoretic analysis. We also noted that emerging modes of warfare, such as mosaic warfare, could be analysed by the application of game theory. Therefore, this review can potentially result in more game-theoretic approaches in modelling such modes of warfare. Furthermore, we have highlighted that the citation network within this field is very sparse: that is, cross-pollination of ideas among various researchers who use game theory in defence applications is rare. This review might serve as a catalyst for collaboration and cross-pollination among researchers in this field. Most importantly, however, this review serves to highlight the utility of game theory in defence applications to defence scientists who have hitherto not used game theory, and will therefore result in introducing a fresh set of tools to defence scientists that they can apply in their research.

As the world deals with emerging challenges to peace and stability, the future of humanity depends on our ability to solve problems peacefully. While this is a lofty goal to achieve, the projection of power is decidedly better than an actual armed conflict which will be very costly at many levels, and game theory could indeed play a part in deciding some of the ‘soft conflicts’ which could play out in the coming years and decades. As the focus on defence strategies and capabilities are likely to increase in the coming years, game theory can serve as an additional tool that defence scientists can use at many levels of abstraction to solve deployment, sensing, tracking, and resource allocation problems.

## Figures and Tables

**Table 1:** Classification System used in this review.

Focus Area	Command and Control Warfare				
Mode	Traditional (T)			Modern (M)	
Warfare Domain	Land (L)	Sea (S)	Air (A)	Cyber (C)	Space (Sp)
Warfare Type	Resource Allocation Warfare (RAW) or Information Warfare (IW) or Weapon Control Wafare (WCW) or Adversary Monitoring Warfare (AMW)				
Game Theory Categorisation 1	Non-Cooperative (NCo) or Cooperative (Co)				
Game Theory Categorisation 2	Sequential (Seq) or Simultaneous (Sim)				
Game Theory Categorisation 3	Discrete (D) or Continuous (C)				
Game Theory Categorisation 4	Zero Sum (ZS) or Non-Zero Sum (NZS)				
Number of Players	2 player (2P) or 3-player (3P) or More than three-player (NP)				

**Table 2:** Classification of papers in the niche of game-theoretic applications in defence, using Table 1. A total of 29 papers described here are classified.

Title	Authors	Classification Code Using Table 1
Game Theoretic analysis of adaptive radar jamming	Bachmann et al.	T-N-IW-NCo-Sim- -D-ZS-2P
Target selection for tracking in multifunction radar networks: Nash and Correlated equilibria	Bogdanovic et al.	T-A-AMW-NCo- -Sim-D-ZS-NP
Power allocation game between a radar network and multiple jammers	Deligiannis et al.	T-A-IW-NCo-Sim-D-ZS-NP
Strategies for defending a coastline against multiple attackers	Garcia et al.	T-A-IW-NCo-Sim-C-ZS-3P
A game theory approach to target tracking in sensor networks	Gu et al.	T-L-IW-NCo-Sim-D-ZS-NP
Joint Power allocation and beamforming between a multi-static-radar and jammer based on game theory	He et al.	T-A-IW-NCo-Sim-D-ZS-2P
Game theoretic situation and transmission in unattended ground sensor networks: a correlated equilibrium approach	Krishnamurthy et al.	T-L-(RAW & AMW)- -NCo-Sim-D-ZS-NP
Network enabled missile deflection: games and correlated equilibrium	Maskery et al. 2007 a	T-N-(RAW & IW) -Co-Sim-D-NZS-NP
Decentralised algorithms for netcentric Force Protection against anti-ship missiles	Maskery et al. 2007 b	T-N-(RAW & IW)- -Co-Sim-D-NZS-NP
Search and Screening	Koopman	T-(LSA)-AMW-NCo- -Slt-D-ZS-NP
Optimal Strategy for Target Protection with a defender in the pursuit-evasion scenario	Qilong et al.	T-A-WCW- -NCo-Sim-C-ZS-3P
Differential game theory with applications to missiles and autonomous systems guidance	Faruqi	T-A-(AMW,WCW)- -NCo-Slt-C-ZS- -(2P,3P,NP)
A game theoretical interceptor guidance law for ballistic missile defence.	Shinar et al.	T-A-(AMW,WCW)- -NCo-Slt-C-ZS-2P
Pursuit-Evasion games: a tractable framework for anti-jamming in aerial attacks	Parras et al.	T-A-(WCW,IW) -NCo-Slt-C-ZS-2P
A simple game theoretic approach to suppression of enemy defences and other time-critical target analyses	Hamilton et al.	T-A-(RAW,WCW)- -NCo-Slt-D-ZS-2P
Choosing What to Protect: Strategic Defence allocation against an unknown attacker	Bier et al.	T-L-RAW-NCo- -Slt-D-ZS-2P
Considerations on Optimal Resource allocation in avation security	Cioaca	M-C-RAW-NCo -Slt-D-ZS-2P
Horsemen and the empty city: A game theoretic examination of deception in Chinese military legend	Cotten et al.	T-L-RAW- -NCo-Slt-D-ZS-2P
An Economic Theory of Destabilisation War	Gries et al.	(T,M)-(IW,RAW)- -NCo-Slt-D-ZS-NP
Game theoretic approach towards network security: A review	Litti	M-C-IW-NCo-Slt-D-ZS-2P
Automating cyber defence responses using attack-defence trees and game theory	Jhawar et al.	M-C-RAW-NCo- -Slt-D-ZS-2P
From individual decisions from experience to behavioural game theory	Gonzalez	M-C-IW-NCo- -Slt-D-ZS-2P
Game Theoretic Approaches to Attack Surface Shifting. Moving Target Defense II	Manadhata	M-C-IW-NCo- -Slt-D-ZS-2P
Improving reliability through Multi-Path routing and Link Defence: An Application of Game Theory to Transport	Kanturska et al.	M-C-RAW- -NCo-Slt-D-ZS-2P
Game Theoretic and Reliability’ models in counter-terrorism and security	Bier et al.	M-C-RAW- -NCo-Slt-D-ZS-2P
Changing behaviours in defence acqusition: a game theory approach	Gardener et al.	(T,M)- -(L,A,S,C,Sp)- -IW-NCo- -Slt-D-ZS-NP
Joint Transmit Power and Bandwidth Allocation for Cognitive Satellite Network based on Bargaining Game Theory	Zhong et al.	M-Sp-RAW-Co- -Slt-D-NZS-NP
The Research and Simulation of a Satellite Routing Algorithm based on Game Theory	Qiao et al.	M-Sp-WCW- -NCo-Slt-D-ZS-NP
A survey of multiple unmanned vehicles formation control and coordiation. Normal and fault situations	Zhang et al.	T-A-WCW-Co- -Slt-D-NZS-NP

**Table 3:** Google Scholar citation counts of the reviewed papers. Google Scholar citation data was accessed on 18 December 2021.

Paper	Country	Google Scholar Citation Count
Game Theoretic analysis of adaptive radar jamming (Bachmann et al.)	Australia	**53**
Target selection for tracking in multifunction radar networks: Nash and Correlated equilibria (Bogdanovic et al.)	Netherlands	**10**
Power allocation game between a radar network and multiple jammers (Deligiannis et al.)	UK	**32**
Strategies for defending a coastline against multiple attackers (Garcia et al.)	US	**7**
A game theory approach to target tracking in sensor networks (Gu et al.)	UK	**72**
Joint Power allocation and beamforming between a multi-static-radar and jammer based on game theory (He et al.)	China	**3**
Game theoretic situation and transmission in unattended ground sensor networks: a correlated equilibrium approach (Krishnamurthy et al.)	US	**4**
Network enabled missile deflection: games and correlated equilibrium (Maskery et al. 2007 a)	US	**9**
Decentralised algorithms for netcentric Force Protection against anti-ship missiles (Maskery et al. 2007 b)	US	**18**
Search and Screening (Koopman)	US	**8**
Optimal Strategy for Target Protection with a defender in the pursuit-evasion scenario (Qilong et al.)	China	**1**
Differential game theory with applications to missiles and autonomous systems guidance (Faruqi)	Australia	**7**
A game theoretical interceptor guidance law for ballistic missile defence (Shinar et al.)	Israel	**34**
Pursuit-Evasion games: a tractable framework for anti-jamming in aerial attacks (Parras et al.)	Spain	**2**
A simple game theoretic approach to suppression of enemy defences and other time-critical target analyses (Hamilton et al.)	US	**17**
Choosing What to Protect: Strategic Defence allocation against an unknown attacker (Bier et al.)	US	**388**
Considerations on Optimal Resource allocation in avation security (Cioaca)	Romania	**3**
Horsemen and the empty city: A game theoretic examination of deception in Chinese military legend (Cotten et al.)	US	**9**
An Economic Theory of Destabilisation War (Gries et al.)	Germany	**3**
Game theoretic approach owards network security: A review (Litti)	India	**7**
Automating cyber defence responses using attack-defence trees and game theory (Jhawar et al.)	Netherlands	**3**
From individual decisions from experience to behavioural game theory (Gonzalez)	US	**9**
Game Theoretic Approaches to Attack Surface Shifting. Moving Target Defense II (Manadhata)	US	**70**
Improving reliability through Multi-Path routing and Link Defence: An Application of Game Theory to Transport (Kanturska et al.)	UK	**13**
Game Theoretic and Reliability models in counter-terrorism and security (Bier et al.)	US	**99**
Changing behaviours in defence acqusition: a game theory approach (Gardener et al.)	UK	**20**
Joint Transmit Power and Bandwidth Allocation for Cognitive Satellite Network based on Bargaining Game Theory (Zhong et al.)	China	**13**
The Research and Simulation of a Satellite Routing Algorithm based on Game Theory (Qiao et al.)	China	**2**
A survey of multiple unmanned vehicles formation control and coordiation. Normal and fault situations (Zhang et al.)	Canada	**98**

**Table 4:** Citations among reviewed papers according to Google Scholar. Google Scholar citation data was accessed on 18 December 2021. It is observable from this table that citing each other’s work is extremely rare in the field.

Paper	Country	Cited By
Game Theoretic analysis of adaptive radar jamming (Bachmann et al.)	Australia	Bogdanovic et al., Deligiannis et al.
Target selection for tracking in multifunction radar networks: Nash and Correlated equilibria (Bogdanovic et al.)	Netherlands	He et al.
Power allocation game between a radar network and multiple jammers (Deligiannis et al.)	UK	He et al.
Strategies for defending a coastline against multiple attackers (Garcia et al.)	US	None
A game theory approach to target tracking in sensor networks (Gu et al.)	UK	None
Joint Power allocation and beamforming between a multi-static-radar and jammer based on game theory (He et al.)	China	None
Game theoretic situation and transmission in unattended ground sensor networks: a correlated equilibrium approach (Krishnamurthy et al.)	US	None
Network enabled missile deflection: games and correlated equilibrium (Maskery et al. 2007 a)	US	Bachmann et al.
Decentralised algorithms for netcentric Force Protection against anti-ship missiles (Maskery et al. 2007 b)	US	Bachmann et al.
Search and Screening (Koopman)	US	None
Optimal Strategy for Target Protection with a defender in the pursuit-evasion scenario (Qilong et al.)	China	None
Differential game theory with applications tomissiles and autonomous systems guidance (Faruqi)	Australia	None
A game theoretical interceptor guidance law for ballistic missile defence (Shinar et al.)	Israel	Faruqi
Pursuit-Evasion games: a tractable framework for anti-jamming in aerial attacks (Parras et al.)	Spain	None
A simple game theoretic approach to suppression of enemy defences and other time-critical target analyses (Hamilton et al.)	US	None
Choosing What to Protect: Strategic Defence allocation against an unknown attacker (Bier et al. a)	US	None
Considerations on Optimal Resource allocation in avation security (Cioaca)	Romania	None
Horsemen and the empty city: A game theoretic examination of deception in Chinese military legend (Cotten et al.)	US	None
An Economic Theory of Destabilisation War (Gries et al.)	Germany	None
Game theoretic approach towards network security: A review (Litti)	India	None
Automating cyber defence responses using attack-defence trees and game theory (Jhawar et al.)	Netherlands	None
From individual decisions from experience to behavioural game theory (Gonzalez)	US	None
Game Theoretic Approaches to Attack Surface Shifting. Moving Target Defense II (Manadhata)	US	None
Improving reliability through Multi-Path routing and Link Defence: An Application of Game Theory to Transport (Kanturska et al.)	UK	None
Game Theoretic and Reliability models in counter-terrorism and security (Bier et al. b)	US	Bier et al. a
Changing behaviours in defence acqusition: a game theory approach (Gardener et al.)	UK	None
Joint Transmit Power and Bandwidth Allocation for Cognitive Satellite Network based on Bargaining Game Theory (Zhong et al.)	China	None
The Research and Simulation of a Satellite Routing Algorithm based on Game Theory (Qiao et al.)	China	None
A survey of multiple unmanned vehicles formation control and coordiation. Normal and fault situations (Zhang et al.)	Canada	None
